# Association of metabolic obesity phenotypes with risk of overall and site-specific cancers: a systematic review and meta-analysis of cohort studies

**DOI:** 10.1038/s41416-024-02857-7

**Published:** 2024-09-24

**Authors:** Yahya Mahamat-saleh, Dagfinn Aune, Heinz Freisling, Sheetal Hardikar, Rola Jaafar, Sabina Rinaldi, Marc J. Gunter, Laure Dossus

**Affiliations:** 1 Nutrition and Metabolism Branch, International Agency for Research on Cancer, Lyon, France; 2 Department of Epidemiology and Biostatistics, School of Public Health, Imperial College London, London, United Kingdom; 3 Department of Research, Cancer Registry of Norway, Norwegian Institute of Public Health, Oslo, Norway; 4 Department of Nutrition, Oslo New University College, Oslo, Norway; 5 Department of Population Health Sciences, University of Utah, Salt Lake City, Utah USA; 6 Huntsman Cancer Institute, University of Utah, Salt Lake City, Utah USA

**Keywords:** Risk factors, Predictive markers, Cancer epidemiology

## Abstract

**Background:**

Adiposity is a known risk factor for certain cancers; however, it is not clear whether the risk of cancer differs between individuals with high adiposity but different metabolic health status. The aim of this systematic literature review and meta-analysis of cohort studies was to evaluate associations between metabolic obesity phenotypes and overall and site-specific cancer risk.

**Methods:**

PubMed and Embase databases were used to identify relevant cohort studies up to the 6th of June 2023. Random-effects models were used to estimate summary relative risks (SRRs) and 95% confidence intervals (CIs) for the association between metabolic obesity phenotypes and cancer risk. Certainty of evidence was assessed using the Cochrane methods and the GRADE tool. This study is registered with PROSPERO, number CRD42024549511.

**Results:**

A total of 15,556 records were screened, and 31 publications covering 15 unique cohort studies were included in this analysis. Of these studies, 22 were evaluated as being at low risk of bias and 9 at moderate risk of bias. Compared to metabolically healthy normal-weight individuals (MHNW), metabolically unhealthy overweight/obese (MUOW/OB) individuals had a higher risk of overall (SRR = 1.21, 95% CI = 1.02–1.44, *n* = 3 studies, high certainty) and obesity-related cancers (SRR = 1.42, 95% CI = 1.15–1.74, *n* = 3, very low certainty). Specifically, MUOW/OB individuals were at higher risk of cancers of the postmenopausal breast (SRR = 1.32, 95% CI = 1.17–1.48, *n* = 7, low certainty), colorectum (SRR = 1.24, 95% CI = 1.16–1.31, *n* = 6, moderate certainty), endometrium (SRR = 2.31, 95% CI = 2.08–2.57, *n* = 4, high certainty), thyroid (SRR = 1.42, 95% CI = 1.29–1.57, *n* = 4, moderate certainty), kidney (SRR = 1.71, 95% CI = 1.40–2.10, *n* = 3, low certainty), pancreas (SRR = 1.35, 95% CI = 1.24–1.47, *n* = 3, high certainty), liver (SRR = 1.81, 95% CI = 1.36–2.42, *n* = 2, moderate certainty), gallbladder (SRR = 1.42, 95% CI = 1.17–1.73, *n* = 2, high certainty), bladder (SRR = 1.36, 95% CI = 1.19–1.56, *n* = 2, moderate certainty), and stomach (SRR = 1.50, 95% CI = 1.12–2.01, *n* = 2, high certainty). In addition, we found elevated risks of most of these cancers among individuals classified as MUNW and MHOW/OB phenotypes compared to those with MHNW phenotype. Our stratified analyses according to metabolic obesity phenotypes suggested that the elevated risks of some cancers were stronger in individuals with MUOW/OB versus those with MHOW/OB or MUNW phenotypes.

**Conclusion:**

These findings suggest that both higher adiposity and metabolic dysfunction were independently associated with increased risk of several cancers, with the strongest associations generally observed among those with both metabolic dysfunction and obesity.

## Introduction

Modifiable lifestyle risk factors such as smoking, alcohol, unhealthy diet, obesity, and lack of physical activity, have been estimated to cause at least 40% of cancers [1–3], and it has been suggested that about 4–8% of cancer cases are attributed to excess body weight [1–4]. Although excess body fatness is a well-established risk factor for at least 13 different cancer sites [5, 6], potential biological mechanisms underlying the adiposity–cancer relationship are not yet fully understood. Excess body fat may increase cancer risk through alterations in the metabolism of endogenous hormones, chronic low-grade inflammation, and insulin resistance or hyperinsulinemia [5, 7]. Studies reported that insulin resistance plays a more significant role in the obesity-cancer association than oestradiol levels and inflammation [8–10]. However, despite excess body weight and hyperinsulinemia having been reported to be associated with an increased risk of cancer, it is unclear whether the risk of obesity-related cancers differs between individuals who are metabolically healthy (i.e., normal insulin sensitivity) with overweight/obesity (MHOW/OB), those with metabolically unhealthy (i.e., insulin resistance) overweight/obesity phenotypes (MUOW/OB) and those with metabolically unhealthy normal weight (MUNW).

In the last decade, a growing body of research has examined the association between metabolic obesity phenotypes—defined using a combination of adiposity and metabolic dysfunction parameters—and obesity-related outcomes [11–14]. Interestingly, several studies reported that MU individuals, defined as having elevated levels of homoeostasis model assessment of insulin resistance (HOMA-IR) index, fasting insulin, C-peptide, or having more than one metabolic abnormality, were at greater risk of developing colorectal, breast, pancreatic, and bladder cancers, regardless of whether they were normal weight or overweight/obese when compared with individuals who are metabolically healthy and normal weight (MHNW) [15–20]. In contrast, although some studies found that individuals who are overweight or obese and MH were also at elevated risk of postmenopausal breast, endometrial, pancreas, and kidney cancers compared to MHNW phenotype [21, 22], accumulating evidence suggests that individuals classified as MHOW/OB were not at elevated risk of these diseases [16, 19, 23, 24]. A subsequent analysis within the UK Biobank found that overweight/obese individuals with metabolic abnormalities had a lower risk of prostate cancer compared with normal-weight individuals with no metabolic abnormalities [21], whereas another study in the National Health Checkup database reported that metabolic syndrome was associated with an increased risk of prostate cancer among individuals with overweight and obesity [17]. Globally, the findings from previous studies have not been consistent, as some studies reported an elevated risk of certain cancer types among MHOW/OB individuals [21, 22], while others found no clear association [16, 19, 23, 24]. A meta-analysis of seven cohort studies reported a higher risk of cancer among individuals with MHOW/OB compared to individuals with MHNW phenotypes [25]. However, the authors of this review provided a pooled risk estimate of the associations by combining different cancer types and did not consider individuals with MU phenotypes. Another meta-analysis, based on seven studies published until June 2021, reported a higher risk of colorectal cancer among individuals classified as MUNW, those with MHOW/OB, and those with MUOW/OB compared with MHNW individuals [26]. A more recent meta-analysis of 11 cohort studies has been published, which focused solely on comparing cancer risk between adults with MHOW/OB and MUOW/OB [27]. However, it is not clear whether individuals with MHOW/OB, MUNW, or MUOW/OB phenotypes compared to MHNW individuals are at a higher risk of overall and site-specific cancer, as these previous meta-analyses did not perform separate analyses.

Since the publication of these three meta-analyses, several cohort studies have been published on the association between metabolic obesity phenotypes and cancer risk, and the strength of the associations differed greatly between these studies [18, 19, 28–34]. Furthermore, previous meta-analyses were limited by the lack of subgroup analyses stratified by study characteristics such as the risk of bias, and the criteria used to define metabolic health status [25–27]. To improve our understanding and identify individuals at high risk among those with high body fatness, it is essential to explore cancer heterogeneity, which has been somewhat limited in previous research—both in terms of cancer subtypes and of metabolic obesity phenotypes exposure.

In this study, we conducted a systematic review and meta-analysis of published cohort studies to investigate the association of metabolically obesity phenotypes with cancer risk overall and for obesity-related cancers and specific cancer sites, to clarify the strength of these associations, and to explore potential sources of heterogeneity between studies.

## Materials and methods

### Search strategy

This systematic review was registered at PROSPERO and accepted for inclusion in June 2024 (Registration ID Number CRD42024549511). We followed the guidelines of the Preferred Reporting Items for Systematic Reviews and Meta-Analyses (PRISMA) statement and Meta-Analysis of Observational Studies in Epidemiology [35]. PubMed (MEDLINE) and Embase databases were systematically searched to identify relevant articles published in English up to June 06, 2023. The search terms and algorithm used are detailed in Supplementary information. We followed the standard criteria for reporting meta-analyses of observational studies [36].

### Study selection and inclusion criteria

In this meta-analysis, we included cohort studies (retrospective, prospective, and nested case-control studies within cohort studies) that examined the association between metabolic obesity phenotypes (MHOW/OB, MUNW, or MUOW/OB vs MHNW phenotypes) and the risk of cancer, overall or for site-specific cancers. Estimates of the relative risk (RR) (such as hazard ratios, risk ratios, or odds ratios) and the 95% confidence intervals (CIs) had to be available in each included publication. Case-control studies, cross-sectional studies, case reports, reviews, editorials, and studies published as conference abstracts only were excluded from this meta-analysis. If several publications were published using the same study cohort, the one with the largest sample size was retained. All relevant studies were imported into Reference Manager for screening. Two reviewers (YM-S and RJ) performed screening by reviewing titles, abstracts, and keywords for relevance to metabolic health or obesity, and cancer. The full text of the selected articles was then retrieved to assess their eligibility. Any discrepancies were resolved by discussion with a third reviewer (DA).

### Data collection

Relevant information was extracted by one investigator (YM-S), and the accuracy of the extractions was checked by another investigator (DA). The following data were collected from each individual publication: the last name of the first author, publication year, country where the research was conducted, study design, study description or name, duration of follow-up, sample size (such as the number of cases and non-cases for nested case-control studies, and cases and total participants for cohort studies), definition of MU status, cancer type, exposure categories, risk estimates with corresponding 95% CIs, and adjustment factors. Additionally, we extracted details regarding the assessment of metabolic health status employed by each study.

### Quality assessment and risk of bias

To assess the quality of the included studies and their potential risk of bias, we used the Cochrane risk of bias tool for non-randomised studies of interventions (ROBINS-I) tool [37]. The tool comprises seven domains: bias due to confounding, bias in selection of study participants, bias in exposure measurement, bias due to misclassification of exposure during follow-up, bias due to missing data, bias in measurement of outcomes, and bias in selection of reported results. The evaluation was independently performed by two reviewers (YM-S and RJ).

### Certainty of evidence

In addition to the assessment of risk of bias, certainty of evidence of pooled associations was evaluated using the Grading of Recommendations, Assessment, Development, and Evaluations (GRADE) approach [38]. This method assesses the quality and the strength of research evidence reflecting the Bradford Hill criteria for causation, which takes into account the within-study risk of bias, inconsistency, indirectness and imprecision between the studies, publication bias, large magnitude of effect, and the impact of residual confounding. Briefly, the certainty of evidence level is considered as “high” for all cohort studies. However, the certainty of evidence is downgraded (up to two levels) unless the study design reduces confounding, selection, and information bias as evaluated by ROBINS-I. The certainty of evidence is rated as “not likely” for risk of bias because all studies are evaluated as low/moderate risk of bias. In addition, indication for inconsistency (as measured by the similarity of the point estimates, and statistical tests, such as *I*^2^), indirectness (e.g., substantial differences in population or exposure), imprecision (wide 95% confidence interval and/or small number of events), and publication bias (Egger’s test) can lead to a downgrading, while large effects (SRR > 2.0) gradient can lead to an upgrading. The certainty of evidence was rated as high, moderate, low, or very low. A high certainty of evidence indicates a strong likelihood that the inclusion of additional studies will have minimal impact on the estimated effect, whereas a very low certainty of evidence implies a high probability that future studies will significantly alter the obtained results.

### Data analysis

All statistical analyses were conducted using R and Stata software version 15.1 and 17. In this study, separate meta-analyses were conducted for the three main exposures of interest: MHOW/OB vs MHNW phenotype, MUNW vs MHNW phenotype, and MUOW/OB vs MHNW phenotype. The analyses were further performed separately for individuals with overweight and obesity to see whether there was a difference in effect size between overweight and obesity. We calculated summary relative risks (SRRs) and 95% CIs to assess the association between metabolic obesity phenotypes, and cancer risk, both overall and by specific cancer sites. The average of the natural logarithm of the RRs was estimated, and the RR from each study was weighted using random effects weights [39]. For studies that reported results separately for specific subgroups of exposure (e.g., MHOW and MHOB or MUOW and MUOB) [21, 23, 32–34, 40, 41], we combined the results using a fixed-effects model to obtain an overall estimate for use in the meta-analysis [42]. For studies that report results separately for multiple cancer types [21], we defined obesity-related cancers using a fixed-effects model that combines 12 different cancer sites concluded with strong evidence of being related to obesity as previously reported in the Third Expert Report of the World Cancer Research Fund including cancers of the mouth/pharynx/larynx, oesophagus, stomach, pancreas, gallbladder, liver, colorectum, postmenopausal breast, endometrium, ovaries, prostate, and kidney [6]. In a study conducted within the Framingham Heart Study, obesity-related cancer was reported as combination of postmenopausal breast cancer, female reproductive (i.e., cervical, endometrial, and uterine), colon, liver, gallbladder, pancreas, kidney, and oesophageal adenocarcinoma [24], whereas in a data from the Metabolic Syndrome and Cancer Project, it was reported as combination of 12 different cancer sites [34].

Statistical heterogeneity between studies was quantitatively assessed by the Cochran Q test and the *I*^2^ statistic [43]. *I*^2^ is a measure of how much of the heterogeneity is due to between-study variation. *I*^2^ values of 25%, 50%, and 75% were considered to indicate low, moderate, and high heterogeneity, respectively. Small-study effects, such as publication bias, were visually assessed by examining the funnel plots for asymmetry and applying Egger’s test [44]. The results were considered to indicate potential small-study bias when p-values were <0.10.

To investigate potential sources of heterogeneity, we conducted subgroup analyses by study characteristics, such as geographic location, duration of follow-up, definition of metabolic health status, and risk of bias, when more than 5 studies were identified for one association. Between-subgroup differences in risk estimates were examined using meta-regression analysis. Sensitivity analyses, excluding one study at a time, were conducted to clarify whether the results were driven by one large study or a study with an extreme result.

## Results

### Literature search and characteristics of included studies

The results of the literature search are shown in Fig. S1. In total, 15,556 records were identified in MEDLINE and in EMBASE. After the exclusion of duplicates, the titles and abstracts from a total of 15163 publications were screened, and out of these, 393 full-text articles were retrieved and assessed for inclusion. Of these, 362 records were excluded due to study design or publication type (abstract, letter, note, commentary, review, or meta-analysis), lack of risk estimates, or irrelevant outcome, exposure, and data. In total, we included 31 publications from 15 unique cohort studies on the associations between metabolic obesity phenotypes and cancer risk that met all the inclusion criteria for the meta-analysis [15–24, 28–34, 40, 41, 45–56]. Of the included articles, 15 publications were from Asia, 9 publications from North America, and 7 publications from Europe. All studies were conducted in adult subjects, the age at baseline ranged from 20 to 79 years for the publications that provided an age range. Sample sizes varied widely among publications. The characteristics of the eligible studies are presented in Tables S1–S16.

Out of the 31 publications assessed using the ROBINS-I tool, 22 were evaluated as being at low risk of bias and 9 were considered at moderate risk of bias (Table 1). In this study, risk of bias due to confounding and outcome assessment was low since almost all studies included in this review provided risk estimates that adjusted for potential confounders, and cancer cases were clinically confirmed. However, selection bias was identified at a moderate level in some studies, particularly those conducted among older populations or postmenopausal women. Bias due to exposure assessment was considered moderate in certain studies that used measurement of a single biomarker with a study-specific cut-point to define metabolic health categories, which may not reflect a complete and objective definition of metabolic healthy status. Nevertheless, none of the studies were classified as being at serious risk of bias or at critical risk of bias.

**Table 1:** Risk of bias for the 32 included publications, based on the ROBINS-I tool.

Author, years, country	Bias due to confounding	Bias due to selection of participants	Bias due to exposure assessment	Bias due to misclassification during follow-up	Bias due to missing data	Bias due to measurement of the outcome	Bias due to selective reporting of the results	Overall judgement
Mahamat-saleh et al. [19], Europe	Low	Low	Moderate	Low	Low	Low	Low	Moderate
Gunter et al. [16], USA	Low	Low	Low	Low	Low	Low	Low	Low
Murphy et al. [20], Europe	Low	Low	Moderate	Low	Low	Low	Low	Moderate
Kabat et al. [49], USA	Low	Low	Low	Low	Low	Low	Low	Low
Kliemann et al. [18], Europe	Low	Low	Moderate	Low	Low	Low	Low	Moderate
Moore et al. [24], USA	Low	Moderate	Moderate	Low	Low	Low	Low	Moderate
Park et al. [54], USA	Low	Low	Low	Low	Low	Low	Low	Low
Park et al. [22], Republic of Korea	Low	Low	Low	Low	Low	Low	Low	Low
Kim et al. [50], South Korea	Low	Low	Low	Low	Low	Low	Low	Low
Kim et al. [17], South Korea	Low	Low	Low	Low	Low	Low	Low	Low
Chung et al. [15], Republic of Korea	Low	Low	Low	Low	Low	Low	Low	Low
Hashimoto et al. [48], Japan	Low	Low	Low	Low	Low	Low	Low	Low
Cao et al. [21], UK	Low	Low	Low	Low	Low	Low	Low	Low
Park et al. [54], Republic of Korea	Low	Low	Low	Low	Low	Low	Low	Low
Shin et al. [56], Republic of Korea	Low	Low	Low	Low	Low	Low	Low	Low
Shao et al. [33], UK	Low	Low	Low	Low	Low	Low	Low	Low
Nguyen et al. [31], Republic of Korea	Low	Low	Low	Low	Low	Low	Low	Low
Lin et al. [41], Taiwan	Low	Low	Low	Low	Low	Low	Low	Low
Liang et al. [52], USA	Low	Moderate	Low	Low	Low	Low	Low	Moderate
Lee et al. [28], Republic of Korea	Low	Low	Low	Low	Low	Low	Low	Low
Kwon et al. [51], South Korea	Low	Low	Low	Low	Low	Low	Low	Low
Kabat et al. [23], USA	Low	Moderate	Low	Low	Low	Low	Low	Moderate
Cho et al. [45], Republic of Korea	Low	Low	Low	Low	Low	Low	Low	Low
Arnlov et al. [40], Sweden	Low	Moderate	Low	Low	Low	Low	Low	Moderate
Pasqual et al. [30], USA	Low	Low	Low	Low	Low	Low	Low	Low
Reeves et al. [55], USA	Low	Moderate	Low	Low	Low	Low	Low	Moderate
Dibaba et al. [47], USA	Low	Moderate	Low	Low	Low	Low	Low	Moderate
Cho et al. [46], Republic of Korea	Low	Low	Low	Low	Low	Low	Low	Low
Moon et al. [29], Republic of Korea	Low	Low	Low	Low	Low	Low	Low	Low
Sun et al. [34], Europe	Low	Low	Low	Low	Low	Low	Low	Low
Park et al. [32], Republic of Korea	Low	Low	Low	Low	Low	Low	Low	Low

*ROBINS-I tool* risk of bias in non-randomised studies of interventions tool.

The definition of metabolic health status varied across the studies (Table 2). In most studies, the MU phenotype was defined as having either 1, 2, or 3 metabolic syndrome components based on the criteria of the Adult Treatment Panel III, which includes (1) elevated blood pressure or the use of antihypertensive medication at baseline, (2) hypertriglyceridemia or current use of lipid-lowering medication at baseline, (3) low HDL cholesterol, (4) hyperglycaemia or use of medications for diabetes at baseline, and (5) abdominal obesity. In other studies, elevated levels of glucose or insulin levels, HOMA-IR, C-peptide levels, or C-reactive protein alone was used to define MU phenotype.

**Table 2:** Definition of metabolic health in included studies.

Author, years, country	Abdominal obesity (WC or WHR)	Elevated triglycerides or lipid-lowering medication	Reduced HDL cholesterol or cholesterol-lowering medication use	Abnormal glucose metabolism, HbA1c levels, diabetes, or antidiabetic drug treatment	Elevated blood pressure - hypertension or antihypertensive medication	High C-peptide level	Homoeostatic Model Assessment for Insulin Resistance	C-reactive protein
Mahamat-saleh et al. [19]						✓		
Gunter et al. [16]							✓	
Murphy et al. [20]						✓		
Kabat et al. [49]		✓	✓	✓	✓			
Kliemann et al. [18]						✓		
Moore et al. [24]				✓				
Park et al. [54], USA	✓		✓	✓	✓			
Park et al. [22]	✓	✓	✓	✓	✓			
Kim et al. [50]	✓	✓	✓	✓	✓			
Kim et al. [17]	✓	✓	✓	✓	✓			
Chung et al. [15]	✓	✓	✓	✓	✓			
Hashimoto et al. [48]		✓	✓	✓	✓			
Cao et al. [21]		✓	✓	✓	✓			
Park et al. [54]	✓	✓	✓	✓	✓			
Shin et al. [56]		✓		✓	✓			
Shao et al. [33]		✓	✓	✓	✓			✓
Nguyen et al. [31]	✓	✓	✓	✓	✓			
Lin et al. [41]		✓	✓	✓	✓			
Liang et al. [52]		✓	✓	✓	✓			
Lee et al. [28]		✓		✓	✓			
Kwon et al. [51]		✓	✓	✓	✓		✓	
Kabat et al. [23]	✓	✓	✓	✓	✓			
Cho et al. [45]		✓	✓	✓	✓			
Arnlov et al. [40]	✓	✓	✓	✓	✓			
Pasqual et al. [30]		✓	✓	✓	✓			
Reeves et al. [55]	✓			✓	✓			
Dibaba et al. [47]	✓	✓	✓	✓	✓			
Cho et al. [46]		✓	✓	✓	✓			
Sun et al. [34]		✓		✓	✓			
Moon et al. [29]	✓	✓	✓	✓	✓			
Park et al. [22]	✓	✓	✓	✓	✓			

*HbA1c* haemoglobin, *HDL* high-density lipoprotein, *HOMA-IR* homoeostasis model assessment for insulin resistance, *HT* hypertension, *FBG* fasting blood glucose, *LDL* low-density lipoprotein, *TG* triglycerides, *WC* waist circumference.

### Metabolically unhealthy/overweight or obese

**Fig. 1:**
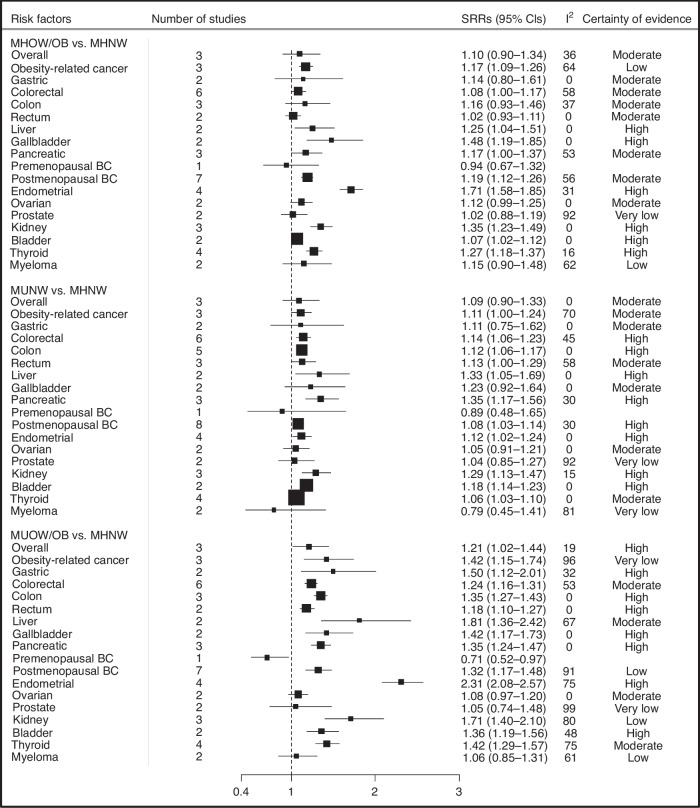
Summary relative risks (SRRs) and 95% CIs for the association between metabolic obesity phenotypes and overall and site-specific cancer risk. BC breast cancer, CI confidence interval, MHNW metabolically healthy normal weight, MHOW/OB metabolically healthy overweight or obese, MUNW metabolically unhealthy normal weight, MUOW/OB metabolically unhealthy overweight or obese, SRR summary relative risks. *I*^2^ is a measure of the proportion of the heterogeneity attributed to between-study variation rather than due to chance.

A total of 27 publications were included in the analysis of MUOW/OB versus MHNW phenotype and risk of overall and site-specific cancers [15–24, 28, 30–34, 40, 41, 45, 46, 48–50, 53–56]. There was high certainty of evidence that individuals classified as MUOW/OB were at a higher risk of overall cancer compared to MHNW individuals (SRR = 1.21, 95% CI = 1.02–1.44, *n* = 3) and there was little evidence of heterogeneity (*I*^2^ = 19%) and no evidence of publication bias (Egger’s test: *P* = 0.91) (Fig. 1). Compared with MHNW individuals, obesity-related cancer risk was higher among those classified as MUOW/OB (SRR = 1.42, 95% CI = 1.15–1.74, *I*^2^ = 96%, *n* = 3, very low certainty of evidence). Specifically, MUOW/OB individuals were at higher risk of cancers of the postmenopausal breast (SRR = 1.32, 95% CI = 1.17–1.48, *I*^2^ = 91%, *n* = 7, low certainty), colorectum (SRR = 1.24, 95% CI = 1.16–1.31, *I*^2^ = 53%, *n* = 6, moderate certainty), colon (SRR = 1.35, 95% CI = 1.27–1.43, *I*^2^ = 0%, *n* = 3, high certainty), rectum (SRR = 1.18, 95% CI = 1.10–1.27, *I*^2^ = 0%, *n* = 2, high certainty), pancreas (SRR = 1.35, 95% CI = 1.24–1.47, *I*^2^ = 0%, *n* = 3, high certainty), gallbladder (SRR = 1.42, 95% CI = 1.17–1.73, *I*^2^ = 0%, *n* = 2, high certainty), bladder (SRR = 1.36, 95% CI = 1.19–1.56, *I*^2^ = 48%, *n* = 2, high certainty), and stomach (SRR = 1.50, 95% CI = 1.12–2.01, *I*^2^ = 32%, *n* = 2, high certainty). In addition, we found that MUOW/OB phenotype was strongly and positively associated with risks of endometrial (SRR = 2.31, 95% CI = 2.08–2.57, *I*^2^ = 75%, *n* = 4, high certainty), thyroid (SRR = 1.42, 95% CI = 1.29–1.57, *I*^2^ = 75%, *n* = 4, moderate certainty), kidney (SRR = 1.71, 95% CI = 1.40–2.10, *I*^2^ = 80%, *n* = 3, low certainty), and liver (SRR = 1.81, 95% CI = 1.36–2.42, *I*^2^ = 67%, *n* = 2, moderate certainty) cancers. Conversely, individuals with the MUOW/OB were not at statistically significant increased risk of ovarian (SRR = 1.08, 95% CI = 0.97–1.20, *I*^2^ = 0%, *n* = 2, moderate certainty) or prostate (SRR = 1.05, 95% CI = 0.74–1.48, *I*^2^ = 99%, *n* = 2, very low certainty) cancers, or myeloma (SRR = 1.06, 95% CI = 0.85–1.31, *I*^2^ = 61%, *n* = 2, low certainty) compared to MHNW individuals, whereas MUOW/OB individuals had a lower risk of premenopausal breast cancer (RR = 0.71, 95% CI = 0.52–0.97, *n* = 1).

**Fig. 2:**
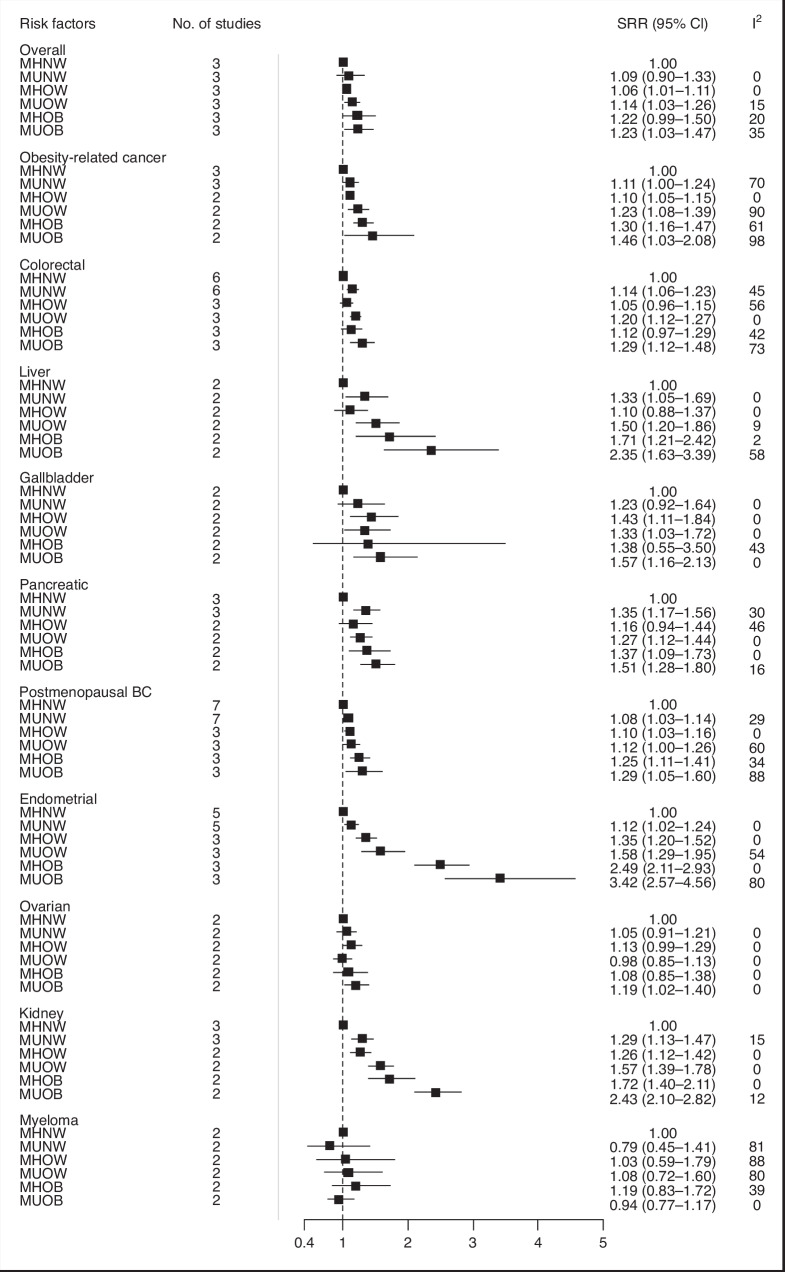
Summary relative risks (SRRs) and 95% CIs for the association between metabolic obesity phenotypes and overall and site-specific cancer risk separately for overweight and obesity. BC breast cancer, CI confidence interval, MHNW metabolically healthy normal weight, MHOB metabolically healthy obese, MHOW metabolically healthy overweight, MUNW metabolically unhealthy normal weight, MUOB metabolically unhealthy obese, MUOW metabolically unhealthy overweight, SRR summary relative risks. *I*^2^ is a measure of the proportion of the heterogeneity attributed to between-study variation rather than due to chance.

When performing separate analyses for overweight and obesity, we found that the MUOW phenotype was associated with increased risks of seven cancers including postmenopausal breast (SRR = 1.12, 95% CI = 1.00–1.26, *I*^2^ = 60%, *n* = 3), colorectal (SRR = 1.20, 95% CI = 1.12–1.27, *I*^2^ = 0%, *n* = 3), endometrial (SRR = 1.58, 95% CI = 1.29–1.95, *I*^2^ = 54%, *n* = 3), pancreas (SRR = 1.27, 95% CI = 1.12–1.44, *I*^2^ = 0%, *n* = 2), kidney (SRR = 1.57, 95% CI = 1.39–1.78, *I*^2^ = 0%, *n* = 2), gallbladder (SRR = 1.33, 95% CI = 1.03–1.72, *I*^2^ = 0%, *n* = 2), and liver (1.50, 95% CI = 1.20–1.86, *I*^2^ = 9%, *n* = 2) cancers (Fig. 2). The MUOB phenotype was positively associated with the risk of eight cancers: postmenopausal breast (SRR = 1.29, 95% CI = 1.05–1.60, *I*^2^ = 88%, *n* = 3), colorectal (SRR = 1.29, 95% CI = 1.12–1.48, *I*^2^ = 73%, *n* = 3), endometrial (SRR = 3.42, 95% CI = 2.57–4.56, *I*^2^ = 80%, *n* = 3), pancreas (SRR = 1.51, 95% CI = 1.28–1.80, *I*^2^ = 16%, *n* = 2), kidney (SRR = 2.43, 95% CI = 2.10–2.82, *I*^2^ = 12%, *n* = 2), gallbladder (SRR = 1.57, 95% CI = 1.16–2.13, *I*^2^ = 0%, *n* = 2), liver (SRR = 2.35, 95% CI = 1.63–3.39, *I*^2^ = 58%, *n* = 2) and ovarian (SRR = 1.19, 95% CI = 1.02–1.40, *I*^2^ = 0%, *n* = 2) cancers (Fig. 2).

### Metabolically unhealthy/normal weight

When comparing MUNW individuals to MHNW individuals, 30 publications were identified [15–24, 28–34, 40, 41, 45–50, 52–56]. We observed that MUNW individuals had a modestly higher risk of obesity-related cancers (SRR = 1.11, 95% CI = 1.00–1.24, *I*^2^ = 70%, *n* = 3, moderate certainty) compared with MHNW (Fig. 1). More specifically, individuals classified as MUNW had increased risks of eight cancers: postmenopausal breast (SRR = 1.08, 95% CI = 1.03–1.14, *I*^2^ = 30%, *n* = 8 studies, high certainty), colorectal (SRR = 1.14, 95% CI = 1.06–1.23, *I*^2^ = 45%, *n* = 6, high certainty), colon (SRR = 1.12, 95% CI = 1.06–1.17, *I*^2^ = 0%, *n* = 5, high certainty), rectal (SRR = 1.13, 95% CI = 1.00–1.29, *I*^2^ = 58%, *n* = 3, moderate certainty), endometrial (SRR = 1.12, 95% CI = 1.02–1.24, *I*^2^ = 0%, *n* = 4, high certainty), thyroid (SRR = 1.06, 95% CI = 1.03–1.10, *I*^2^ = 0%, *n* = 4, moderate certainty), pancreatic (SRR = 1.35, 95% CI = 1.17–1.56, *I*^2^ = 30%, *n* = 3, high certainty), kidney (SRR = 1.29, 95% CI = 1.13–1.47, *I*^2^ = 15%, *n* = 3, high certainty), liver (SRR = 1.33, 95% CI = 1.05–1.69, *I*^2^ = 0%, *n* = 2, high certainty) and bladder (SRR = 1.18, 95% CI = 1.14–1.23, *I*^2^ = 0%, *n* = 2, high certainty) cancers. However, associations between MUNW and overall (SRR = 1.09, 95% CI = 0.90–1.33, *I*^2^ = 0%, *n* = 3, moderate certainty), gallbladder (SRR = 1.23, 95% CI = 0.92–1.64, *I*^2^ = 0%, *n* = 2, moderate certainty), stomach (SRR = 1.11, 95% CI = 0.75–1.62, *I*^2^ = 0%, *n* = 2, moderate certainty), ovarian (SRR = 1.05, 95% CI = 0.91–1.21, *I*^2^ = 0%, *n* = 2, moderate certainty), prostate (SRR = 1.04, 95% CI = 0.85–1.27, *I*^2^ = 92%, *n* = 2, very low certainty), myeloma (SRR = 0.79, 95% CI = 0.45–1.41, *I*^2^ = 81%, *n* = 2, very low certainty), and premenopausal breast (SRR = 0.89, 95% CI = 0.48–1.65, moderate certainty) cancers were not statistically significant when compared to MHNW individuals.

### Metabolically healthy/overweight or obese

In total, 28 publications were included in the analysis of MHOW/OB versus MHNW phenotype and cancer risk [15–24, 28, 30–34, 40, 41, 45, 46, 48–51, 53–56]. Compared to MHNW individuals, those with MHOW/OB were at elevated risk of obesity-related cancer (SRR = 1.17, 95% CI = 1.09–1.26, *I*^2^ = 64%, *n* = 3, low certainty) (Fig. 1). The MHOW/OB phenotype was associated with increased risks of nine cancers: postmenopausal breast (SRR = 1.19, 95% CI = 1.12–1.26, *I*^2^ = 56%, *n* = 7, moderate certainty), colorectal (SRR = 1.08, 95% CI = 1.00–1.17, *I*^2^ = 58%, *n* = 6, moderate certainty), endometrial (SRR = 1.71, 95% CI = 1.58–1.85, *I*^2^ = 31%, *n* = 4, high certainty), thyroid (SRR = 1.27, 95% CI = 1.18–1.37, *I*^2^ = 16%, *n* = 4, high certainty), pancreatic (SRR = 1.17, 95% CI = 1.10–1.37, *I*^2^ = 53%, *n* = 3, moderate certainty), kidney (SRR = 1.35, 95% CI = 1.23–1.49, *I*^2^ = 0%, *n* = 3, high certainty), liver (SRR = 1.25, 95% CI 1.04–1.51, *I*^2^ = 0%, *n* = 2, high certainty), gallbladder (SRR = 1.48, 95% CI = 1.19–1.85, *I*^2^ = 0%, *n* = 2, high certainty) and bladder cancers (SRR = 1.07, 95% CI = 1.02–1.12, *I*^2^ = 0%, *n* = 2, high certainty). However, no statistically significant associations were found with other cancer types when comparing the MHOW/OB phenotype to the MHNW phenotype.

Our separate analyses for overweight and obesity showed that MHOW phenotype was associated with increased risks of four cancers, including postmenopausal breast, endometrial, kidney, and gallbladder cancers, whereas MHOB phenotype was positively and strongly associated with risk of five cancers: postmenopausal breast, endometrial, pancreas, kidney and liver cancers (Fig. 2).

### Certainty of evidence

With regard to the body of evidence, the certainty of evidence was rated as “high” in 23 of 51 risk estimates, “moderate” in 18 of 51 risk estimates, and “low” and “very low” in 5 and 5 of 51 risk estimates, respectively (Table 3). The main reason for the ‘low’ or “very low” certainty was inconsistency, which is attributed to the high heterogeneity between studies and/or publication bias.

**Table 3:** Certainty of evidence for the association between metabolic obesity phenotypes and overall and specific cancer risk.

	Certainty assessment									Relative risk (95% CI)	
Outcome	No of studies	Study design	Risk of bias	Inconsistency	Indirectness	Imprecision (ratio of the upper to lower 95%CI)	Publication bias	Large magnitude of association	Other considerations		Certainty
**MUNW vs. MHNW**											
Overall	3	Cohort study	Not likely	Not likely	Not likely	Serious	Not likely	No upgrade	None	1.09 (0.90–1.33)	Moderate
Postmenopausal BC	8	Cohort study	Not likely	Not likely	Not likely	Not likely	Not likely	No upgrade	None	1.08 (1.03–1.14)	High
Endometrial	4	Cohort study	Not likely	Not likely	Not likely	Not likely	Not likely	No upgrade	None	1.12 (1.02–1.24)	High
Colorectal	6	Cohort study	Not likely	Not likely	Not likely	Not likely	Not likely	No upgrade	None	1.14 (1.06–1.23)	High
Colon	5	Cohort study	Not likely	Not likely	Not likely	Not likely	Not likely	No upgrade	None	1.12 (1.06–1.17)	High
Rectum	3	Cohort study	Not likely	Serious	Not likely	Not likely	Not likely	No upgrade	None	1.13 (1.00–1.29)	Moderate
Thyroid	4	Cohort study	Not likely	Not likely	Not likely	Not likely	Not likely	No upgrade	None	1.06 (1.03–1.10)	High
Pancreatic	3	Cohort study	Not likely	Not likely	Not likely	Not likely	Not likely	No upgrade	None	1.35 (1.17–1.56)	High
Kidney	3	Cohort study	Not likely	Not likely	Not likely	Not likely	Not likely	No upgrade	None	1.29 (1.13–1.47)	High
Gastric	2	Cohort study	Not likely	Not likely	Not likely	Serious	Not likely	No upgrade	None	1.11 (0.75–1.62)	Moderate
Bladder	2	Cohort study	Not likely	Not likely	Not likely	Not likely	Not likely	No upgrade	None	1.18 (1.14–1.23)	High
Prostate	2	Cohort study	Not likely	Very serious	Not likely	Serious	Not likely	No upgrade	None	1.04 (0.85–1.27)	Very low
Ovarian	2	Cohort study	Not likely	Not likely	Not likely	Serious	Not likely	No upgrade	None	1.05 (0.91–1.21)	Moderate
Myeloma	2	Cohort study	Not likely	Very serious	Not likely	Serious	Downgrade	No upgrade	None	0.79 (0.45–1.41)	Very low
Liver	2	Cohort study	Not likely	Not likely	Not likely	Not likely	Not likely	No upgrade	None	1.33 (1.05–1.69)	High
Gallbladder	2	Cohort study	Not likely	Not likely	Not likely	Serious	Not likely	No upgrade	None	1.23 (0.92–1.64)	Moderate
Obesity-related cancer	3	Cohort study	Not likely	Serious	Not likely	Serious	Not likely	No upgrade	None	1.11 (1.00–1.24)	Moderate
Premenopausal BC	1	Cohort study	Not likely	Not likely	Not likely	Serious	Not likely	No upgrade	None	0.89 (0.48–1.65)	
**MHOW/OB vs. MHNW**											
Overall	3	Cohort study	Not likely	Not likely	Not likely	Serious	Not likely	No upgrade	None	1.10 (0.90–1.34)	Moderate
Postmenopausal BC	7	Cohort study	Not likely	Serious	Not likely	Not likely	Not likely	No upgrade	None	1.19 (1.12–1.26)	Moderate
Endometrial	4	Cohort study	Not likely	Not likely	Not likely	Not likely	Not likely	No upgrade	None	1.71 (1.58–1.85)	High
Colorectal	6	Cohort study	Not likely	Serious	Not likely	Not likely	Not likely	No upgrade	None	1.08 (1.00–1.17)	Moderate
Colon	3	Cohort study	Not likely	Not likely	Not likely	Serious	Not likely	No upgrade	None	1.16 (0.93–1.46)	Moderate
Rectum	2	Cohort study	Not likely	Not likely	Not likely	Serious	Not likely	No upgrade	None	1.02 (0.93–1.11)	Moderate
Thyroid	4	Cohort study	Not likely	Not likely	Not likely	Not likely	Not likely	No upgrade	None	1.27 (1.18–1.37)	High
Pancreatic	3	Cohort study	Not likely	Serious	Not likely	Not likely	Not likely	No upgrade	None	1.17 (1.10–1.37)	Moderate
Kidney	3	Cohort study	Not likely	Not likely	Not likely	Not likely	Not likely	No upgrade	None	1.35 (1.23–1.49)	High
Gastric	2	Cohort study	Not likely	Not likely	Not likely	Serious	Not likely	No upgrade	None	1.14 (0.80–1.61)	Moderate
Bladder	2	Cohort study	Not likely	Not likely	Not likely	Not likely	Not likely	No upgrade	None	1.07 (1.02–1.12)	High
Prostate	2	Cohort study	Not likely	Very serious	Not likely	Serious	Not likely	No upgrade	None	1.02 (0.88–1.19)	Very low
Ovarian	2	Cohort study	Not likely	Not likely	Not likely	Serious	Not likely	No upgrade	None	1.12 (0.99–1.25)	Moderate
Myeloma	2	Cohort study	Not likely	Serious	Not likely	Serious	Not likely	No upgrade	None	1.15 (0.90–1.48)	Low
Liver	2	Cohort study	Not likely	Not likely	Not likely	Not likely	Not likely	No upgrade	None	1.25 (1.04–1.51)	High
Gallbladder	2	Cohort study	Not likely	Not likely	Not likely	Not likely	Not likely	No upgrade	None	1.48 (1.19–1.85)	High
Obesity-related cancer	3	Cohort study	Not likely	Serious	Not likely	Not likely	Downgrade	No upgrade	None	1.17 (1.09–1.26)	Low
Premenopausal BC	1	Cohort study	Not likely	Not likely	Not likely	Serious	Not likely	No upgrade	None	0.94 (0.67–1.32)	
**MUOW/OB vs. MHNW**											
Overall	3	Cohort study	Not likely	Not likely	Not likely	Not likely	Not likely	No upgrade	None	1.21 (1.02–1.44)	High
Postmenopausal BC	7	Cohort study	Not likely	Very serious	Not likely	Not likely	Not likely	No upgrade	None	1.32 (1.17–1.48)	Low
Endometrial	4	Cohort study	Not likely	Serious	Not likely	Not likely	Not likely	**Upgrade (strong association)**	None	2.31 (2.08–2.57)	High
Colorectal	6	Cohort study	Not likely	Serious	Not likely	Not likely	Not likely	No upgrade	None	1.24 (1.16–1.31)	Moderate
Colon	3	Cohort study	Not likely	Not likely	Not likely	Not likely	Not likely	No upgrade	None	1.35 (1.27–1.43)	High
Rectum	2	Cohort study	Not likely	Not likely	Not likely	Not likely	Not likely	No upgrade	None	1.18 (1.10–1.27)	High
Thyroid	4	Cohort study	Not likely	Not likely	Not likely	Not likely	Downgrade	No upgrade	None	1.42 (1.29–1.57)	Moderate
Pancreatic	3	Cohort study	Not likely	Not likely	Not likely	Not likely	Not likely	No upgrade	None	1.35 (1.24–1.47)	High
Kidney	3	Cohort study	Not likely	Very serious	Not likely	Not likely	Not likely	No upgrade	None	1.71 (1.40–2.1)	Low
Gastric	2	Cohort study	Not likely	Not likely	Not likely	Not likely	Not likely	No upgrade	None	1.50 (1.12–2.01)	High
Bladder	2	Cohort study	Not likely	Not likely	Not likely	Not likely	Not likely	No upgrade	None	1.36 (1.19–1.56)	High
Prostate	2	Cohort study	Not likely	Very serious	Not likely	Serious	Not likely	No upgrade	None	1.05 (0.74–1.48)	Very low
Ovarian	2	Cohort study	Not likely	Not likely	Not likely	Serious	Not likely	No upgrade	None	1.08 (0.97–1.20)	Moderate
Myeloma	2	Cohort study	Not likely	Serious	Not likely	Serious	Not likely	No upgrade	None	1.06 (0.85–1.31)	Low
Liver	2	Cohort study	Not likely	Serious	Not likely	Not likely	Not likely	No upgrade	None	1.81 (1.36–2.42)	Moderate
Gallbladder	2	Cohort study	Not likely	Not likely	Not likely	Not likely	Not likely	No upgrade	None	1.42 (1.17–1.73)	High
Obesity-related cancer	3	Cohort study	Not likely	Very serious	Not likely	Not likely	Downgrade	No upgrade	None	1.42 (1.15–1.74)	Very low
Premenopausal BC	1	Cohort study	Not likely	Not likely	Not likely	Serious	Not likely	No upgrade	None	0.71 (0.52–0.97)	

*BC* breast cancer, *CI* confidence interval, *MHNW* metabolically healthy normal weight, *MHOW/OB* metabolically healthy overweight or obese, *MUNW* metabolically unhealthy normal weight, *MUOW/OB* metabolically unhealthy overweight or obese.

### Subgroup and sensitivity analyses, and publication bias

Our stratified analyses according to metabolic obesity phenotypes suggested that the elevated risks for five cancers and two cancer subtypes were stronger in individuals with MUOW/OB compared to those with MHOW/OB or MUNW phenotypes: postmenopausal breast (SRR = 1.32, 95% CI = 1.17–1.48), endometrial (SRR = 2.31, 95% CI = 12.08–2.57), colorectal (SRR = 1.24, 95% CI = 1.16–1.31), colon (SRR = 1.35, 95% CI = 1.27–1.43), rectal (SRR = 1.18, 95% CI = 1.10–1.27), thyroid (SRR = 1.42, 95% CI = 1.29–1.57), and bladder cancers (SRR = 1.36, 95% CI = 1.19–1.56) (Figs. 1, S4–S7, S10, 11). There was some indication of heterogeneity between the metabolic obesity phenotypes for these cancer subtypes: postmenopausal breast (*P*_heterogeneity_ = 0.001), endometrial (*P*_heterogeneity_ ≤ 0.0001), colorectal (*P*_heterogeneity_ = 0.02), colon (*P*_heterogeneity_ ≤ 0.0001), rectal (*P*_heterogeneity_ = 0.04), thyroid (*P*_heterogeneity_ ≤ 0.0001), and bladder cancers (*P*_heterogeneity_ = 0.001). Our stratified analyses also suggest that there is a gradual increase in the risk for overall and obesity-related cancers, as well as some specific cancers such as postmenopausal breast, endometrial, and kidney cancer from MUNW, through MHOW and MUOW to MHOB and MUOB by considering MHNW as reference phenotype (Fig. 2).

The positive association between metabolic obesity phenotypes and postmenopausal breast cancer persisted in most subgroup analyses (Table S17). Our meta-regression analysis showed that the definition of MU phenotype, the risk of bias, the geographic location, and the duration of follow-up did not significantly influence the magnitude of the association, particularly for MUOW/OB and MHOW/OB phenotypes. However, heterogeneity between subgroups was observed in analyses of MUNW phenotype stratified by risk of bias with a stronger association for studies with moderate risk of bias compared to those with low risk of bias (*P*_heterogeneity_ = 0.02). The elevated risk of MHOW/OB phenotype with postmenopausal breast cancer was stronger for studies with 5 to <10 vs. ≥10 years duration of follow-up (*P*_heterogeneity_ = 0.002). In contrast, in the analysis of metabolic obesity phenotypes and colorectal cancer, positive associations were consistently observed in almost all subgroup analyses, including analyses by definition of MU phenotype, geographic location, duration of follow-up, and risk of bias (Table S18). Subgroup analyses by study characteristics for the association between metabolic obesity phenotypes and risks of other cancer types could not be performed because of the limited number of studies.

In addition, although influence analyses and publication bias were assessed for each association, we decided to show these results only when more than 4 studies were identified for one association (Figs. S12–S35). We found some evidence of publication bias in the following analyses: MHOW/OB phenotype and obesity-related cancer (*P*_Egger’s test_ = 0.01); MUNW phenotype and myeloma (*P*_Egger’s test_ = 0.09); and MUOW/OB phenotype and thyroid (*P*_Egger’s test_ = 0.03), and obesity-related cancer (*P*_Egger’s test_ = 0.06) (Figs. S12–S23). Finally, in influence analyses excluding one study at a time from each analysis, most of the associations were robust to the influence of individual studies (Figs. S24–S35).

## Discussion

### Principal findings

This meta-analysis reviewed 31 cohort publications on the associations between metabolic obesity phenotypes and risk of overall and site-specific cancers. We found that both metabolic dysfunction and higher adiposity were independently associated with increased risk of several cancers and the strongest associations were in general observed among those with both metabolic dysfunction and obesity. Individuals classified as MUOW/OB had elevated risk of overall and obesity-related cancer compared to MHNW individuals. More specifically, individuals classified as MUOW/OB had increased risks of 10 cancers: postmenopausal breast, endometrium, colorectum, thyroid, pancreatic, gallbladder, bladder, stomach, kidney, and liver. In addition, we found elevated risks of 8 and 9 cancers among individuals classified as MUNW and MHOW/OB phenotypes compared to those with MHNW phenotype, respectively. In addition, our stratified analyses according to metabolic obesity phenotypes suggested that the elevated risks were stronger in individuals with MUOW/OB versus those with MHOW/OB or MUNW phenotypes, with evidence of heterogeneity across metabolic obesity phenotypes exposure for five cancer types.

In subgroup analyses stratified by study characteristics, we observed that the positive association between metabolic obesity phenotypes and postmenopausal breast and colorectal cancers persisted in most subgroup analyses. However, for postmenopausal breast cancer risk, our meta-regression analysis showed that the risk of bias and the duration of follow-up may significantly influence the magnitude of the association with higher risk among MUNW individuals for studies with moderate risk of bias compared to those with low risk of bias, and higher risk among MHOW/OB individuals for studies with 5 to <10 vs. ≥10 years duration of follow-up.

Although there was evidence for some publication bias in the overall analysis, study heterogeneity was high in 9 of 51 risk estimates as measured by the *I*^2^-value, and this persisted in most of the subgroup analyses of postmenopausal breast and colorectal cancer. However, the heterogeneity appeared to be driven to a larger extent by differences in the strength of the associations, than differences in the direction of the associations, as the majority of the included studies reported statistically significant or non-significant positive associations between metabolic obesity phenotypes and cancer risk, and relatively few studies reported risk estimates in the direction of an inverse association.

The certainty of evidence was rated as high in most of the findings and few associations were rated as “very low certainty” which was mainly attributed to the high heterogeneity and/or publication bias.

### Biological mechanisms

A possible mechanism underlying the elevated risk of cancer among MUOW/OB individuals may involve metabolic dysregulation, specifically insulin resistance, hyperinsulinemia, and diabetes [57, 58]. Insulin plays a crucial role in both normal and malignant cells, given that its receptor is commonly found in tumour cells [59]; and insulin resistance can further promote cancer cell growth through its mitogenic and antiapoptotic activities [57, 58]. Studies have demonstrated that adults with high body fatness and elevated insulin levels tend to have abnormal serum concentrations of adipokines, with reduced adiponectin and elevated leptin levels [60, 61], which have been associated with increased risk for adiposity-related cancers [62–64]. In addition, higher circulating levels of insulin could impact cancer risk by regulating levels of sex hormone synthesis and chronic inflammation which in turn increase the risk of obesity-related cancer [8, 10]. It has been reported that individuals with MUOW phenotype had elevated levels of circulating inflammatory cytokines markers, including interleukin (IL-1β), IL-6, IL-8, IL-10, and tumour necrosis factor-α (TNF-α), compared to individuals with MHNW, indicating that both excess body fat and an altered metabolic profile are associated with inflammation [65]. Increasing levels of circulating inflammatory cytokines markers were positively associated with the risk of cancer [66–68].

However, although we found that individuals classified as MHOW/OB had an elevated risk of obesity-related cancer, the mechanisms are still unclear. It is possible that chronic inflammation and sex steroid hormones resulting from excess adipose tissue may explain the higher risk of cancer. These findings suggest that the impact of adiposity on cancer incidence is likely to be at least partly independent of metabolic health. Excess body fatness is linked to elevated levels of hormones and substances from fat cells that promote inflammation, such as leptin, TNF-α, and IL-6, which could potentially stimulate the growth of cancer cells [69, 70]. In fact, despite individuals with MHOW/OB had lower levels of insulin and fewer metabolic abnormalities compared to those with MUOW/OB, they had higher levels of total body and visceral fat compared to the MHNW individuals [14, 71]. In addition, in our meta-analysis, we observed that the elevated risks were stronger in individuals with MUOW/OB versus those with MHOW/OB or MUNW phenotypes, with evidence of heterogeneity across metabolic obesity phenotypes exposure for five cancers and two cancer subtypes: postmenopausal breast, endometrial, colorectal, colon, rectal, thyroid and bladders cancers. Although MHOW/OB phenotype is more commonly found in women than in men and in younger than in older adults [72, 73], a recent meta-analysis showed that sex and age did not influence the association between metabolic obesity phenotypes and cancer risk [25]. The stronger associations among MUOW/OB compared to MHOW/OB phenotype could be attributed to metabolic dysfunction.

### Strengths, limitations and public health implications

The current meta-analysis of cohort studies is one of the largest meta-analyses on the association between metabolic obesity phenotypes and risk of cancer to date and included 31 publications published through June 2023. This is also, to our knowledge, the first meta-analysis that assessed the risk of site-specific cancers. Notable strengths of this meta-analysis include its large sample size that ensured statistical power of findings, the detailed subgroup and sensitivity analyses, and the cohort design of the included studies, reducing the potential for recall bias, selection bias, and reverse causation, which may affect case-control and cross-sectional studies. Although assessment of the causality of these findings using randomised controlled trials design is not possible because we cannot randomise people according to obesity or metabolic health, all included studies were rated low or moderate risk of bias, and certainty of evidence was high in most of the risk estimates. To date, there is no universally accepted standard for defining MU phenotype [74]: most of the included studies defined MU phenotype using the common definition of metabolic syndrome (based on ATP- III components) and few studies used measurement of one biomarker to classify individuals according to their metabolic health status. However, our meta-regression and stratified analyses by the definition of the MH status did not show a difference in these associations. Despite these strengths, several limitations of this review need to be considered. The main limitation is the lack of information on changes in metabolic phenotype and adiposity over time, and our findings did not take into account longitudinal changes in body weights or metabolic markers parameters. Previous studies reported that nearly 30 to 50% of individuals classified as MHOW/OB phenotype at inclusion shifted to a MU phenotype over the follow-up time, whereas about 25 to 30% of individuals classified as MUNW phenotype recovered their metabolic health [75–77]. In addition, although BMI provides the most useful population-level measure of overweight and obesity, it does not differentiate fat tissue from lean tissue and very few studies assessed overweight and obesity using waist circumference, waist-to-hip ratio, or body fat percentage. Subgroup analyses stratified by use of medications, including statins and metformin, duration of metabolic dysfunction, and by lifestyle such as physical activity, were not possible because of the lack of such data from the studies included. Although the GRADE approach is considered an ideal tool for assessing the certainty of a body of evidence, it may be excessively conservative. Also, for some of the current findings that were rated with high certainty and where few studies were included such as pancreas, gallbladder, and stomach, additional studies could potentially alter the results, however, we still consider this less likely as these are cancers with established associations with adiposity [5]. Lastly, for several cancer sites, the number of studies was somewhat limited, thus further studies are needed across less explored cancer sites.

In the context of the increasing prevalence of overweight and obesity [78], which contributes to a large number of cancer cases [1–3, 6], and since some individuals with overweight or obesity do not present any metabolic dysfunction, these findings may have important public health implications. Relying solely on anthropometric parameters to identify individuals at elevated risk of cancer could lead to the exclusion of normal-weight individuals with poor metabolic health; hence, this might underestimate the risk of cancer in individuals with poor metabolic health. Our findings lend support to the importance of assessing metabolic dysfunction status in addition to anthropometric measurement in routine clinical practice.

## Conclusion

Our findings suggest that both metabolic dysfunction and excess body fatness increase the risk of multiple cancers, including postmenopausal breast, colorectal, pancreatic, endometrial, gallbladder, stomach, bladder, liver, kidney, and thyroid cancer. The combination of adiposity and metabolic dysfunction is associated with the strongest increase in risk. Although further cohort studies are needed to investigate changes in metabolic obesity phenotypes over time, these findings highlighted the importance of combining measures of adiposity with indicators of metabolic dysfunction, and this may be useful in identifying individuals at higher risk of cancer in addition to the existing screening practices.

## Supplementary information

Supplementary figures

Supplementary tables

## Data Availability

All data relevant to the study are included in the article or uploaded as supplementary information. Correspondence and requests for materials should be addressed to YM-S.
