# Supplementary material for: Association of metabolic obesity phenotypes with risk of overall and site-specific cancers: a systematic review and meta-analysis of cohort studies
Source: Br J Cancer. 2024 Sep 24;131(9):1480–95. doi: 10.1038/s41416-024-02857-7 (PMC11519895; doi:10.1038/s41416-024-02857-7)
Supplement: Supplementary file 1 — Supplementary figures [file 41416_2024_2857_MOESM1_ESM.docx]

**Supplementary text Search strategy**

**Embase search terms:**

| 1 | (Metabolic or metabolism or metabolically or healthy or unhealthy or Abnormal).ab,ti. | 3468272 | Advanced |
| --- | --- | --- | --- |
| 2 | Metabolic/ or metabolism/ or metabolically/ or healthy/ or unhealthy/ or Abnormal/ | 1464807 | Advanced |
| 3 | (obese or obesity or overweight or BMI or body mass index or waist circumference).ab,ti. | 983938 | Advanced |
| 4 | obese/ or obesity/ or overweight/ or BMI/ or body mass index/ or waist circumference/ | 982406 | Advanced |
| 5 | (Cancer or neoplasm or tumor or tumour).ab,ti. | 4471906 | Advanced |
| 6 | Cancer/ or neoplasm/ or tumor/ or tumour/ | 830014 | Advanced |
| 7 | (Cohort or cohorts or prospective or longitudinal or follow-up or case-control or cross-sectional).af. | 5133991 | Advanced |
| 8 | 1 or 2 | 4601122 | Advanced |
| 9 | 3 or 4 | 1238147 | Advanced |
| 10 | 5 or 6 | 4676837 | Advanced |
| 11 | 7 and 8 and 9 and 10 | 8888 | Advanced |

**PubMed search terms:**

1. "Metabolic" OR "metabolism" OR " Metabolically" OR "healthy" OR "unhealthy" OR "Abnormal”
2. "Obese" OR "obesity" OR "overweight" OR "BMI" OR "body mass index" OR "waist circumstance"
3. "Cancer" OR "neoplasm" OR "tumor" OR "tumour"
4. "Cohort" OR "cohorts" OR "prospective" OR "longitudinal" OR "follow-up" OR "case-control" OR cross-sectional
5. 1 AND 2
6. 5 AND 3 AND 4

**Figure S1**. Flow chart of study selection for a systematic review and meta-analysis of metabolic obesity phenotypes and overall and site-specific cancer risk

**Figure S2.** Summary estimates for the association between metabolic obesity phenotypes and overall cancer risk


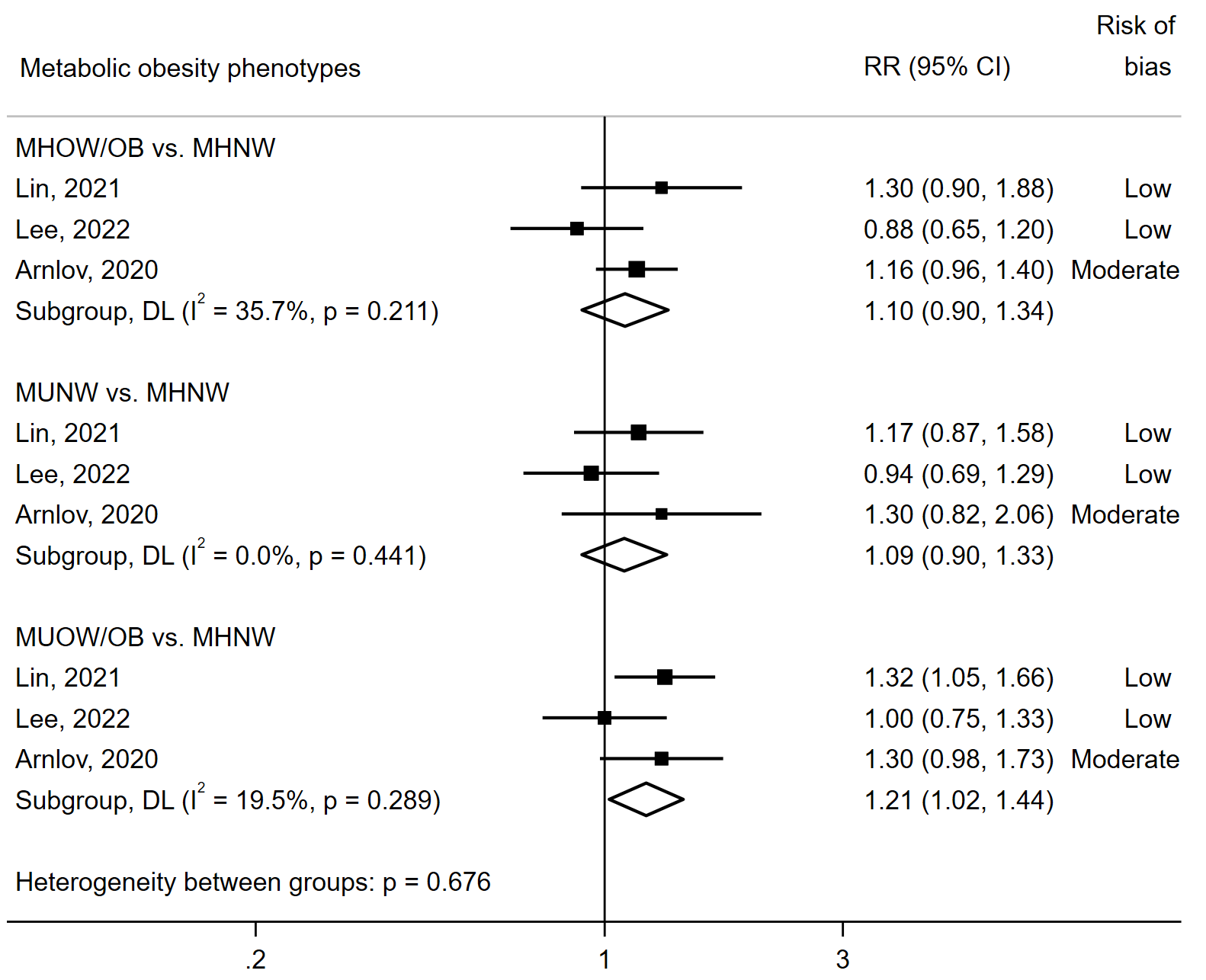


**Figure S3.** Summary estimates for the association between metabolic obesity phenotypes and obesity related-cancer risk


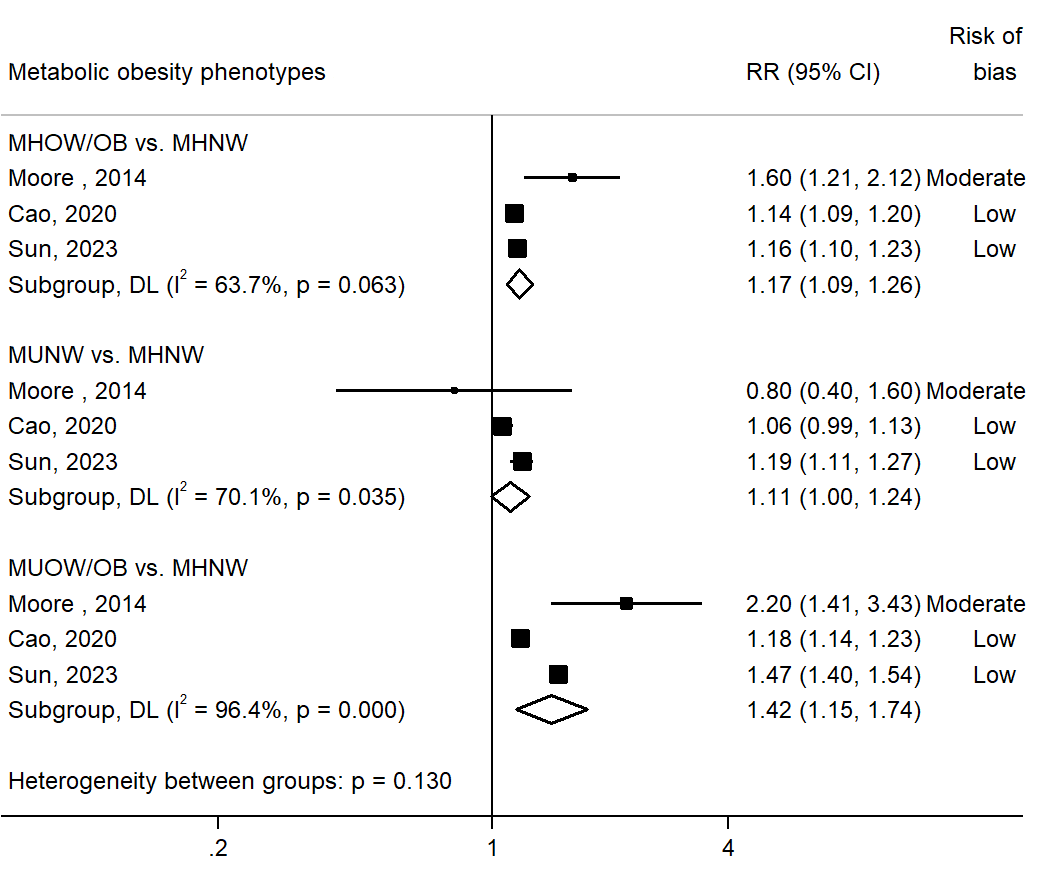


**Figure S4.** SRRs and 95% CIs for the association between metabolic obesity phenotypes and postmenopausal breast cancer risk


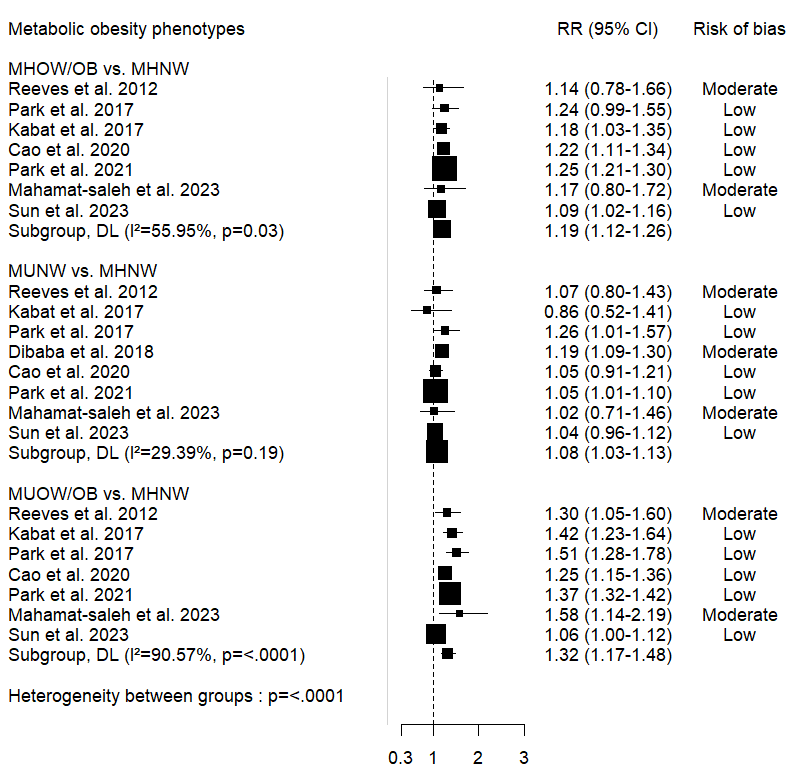


**Figure S5.** SRRs and 95% CIs for the association between metabolic obesity phenotypes and colorectal cancer risk


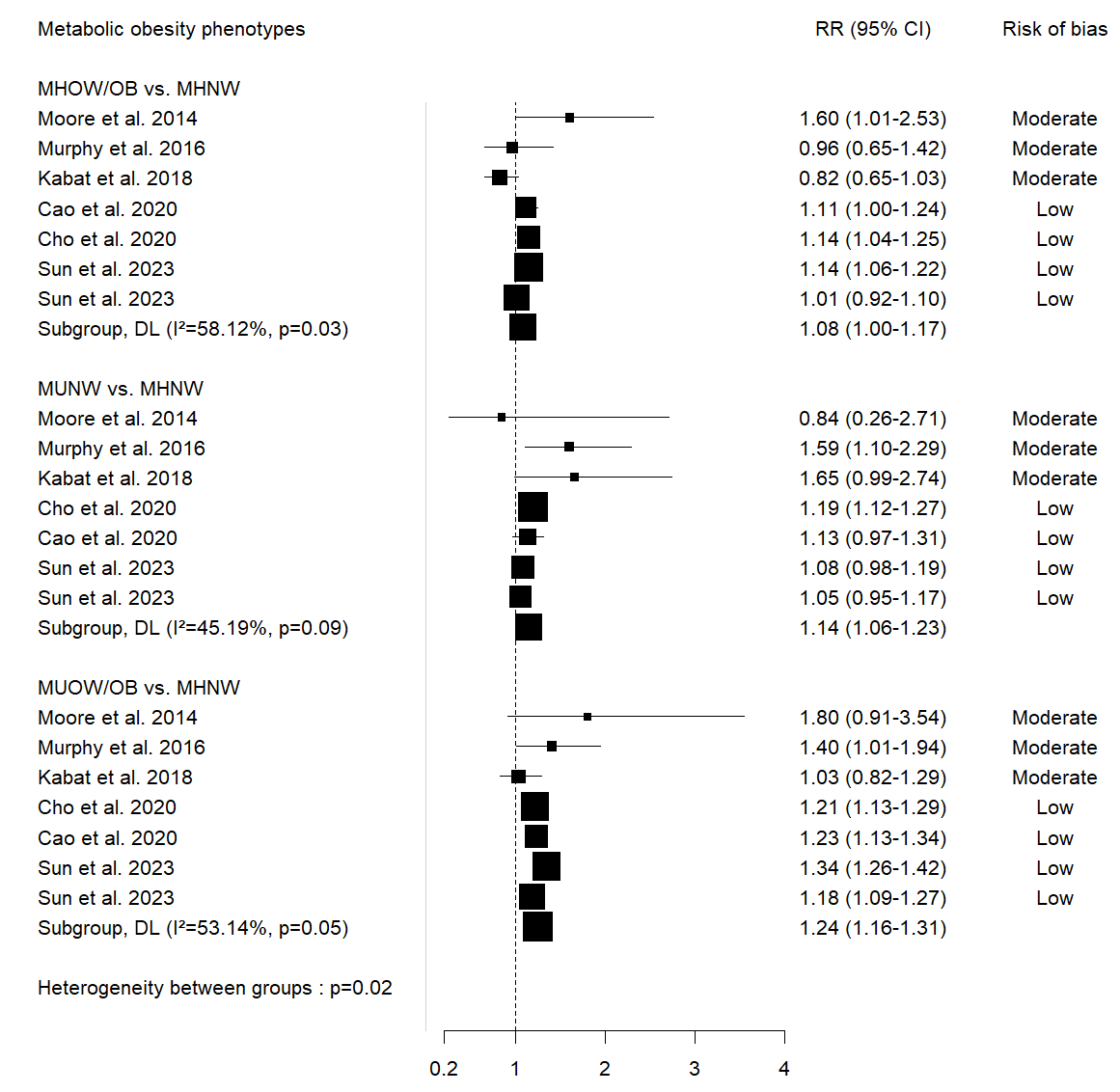


**Figure S6.** Summary estimates for the association between metabolic obesity phenotypes and endometrial cancer risk


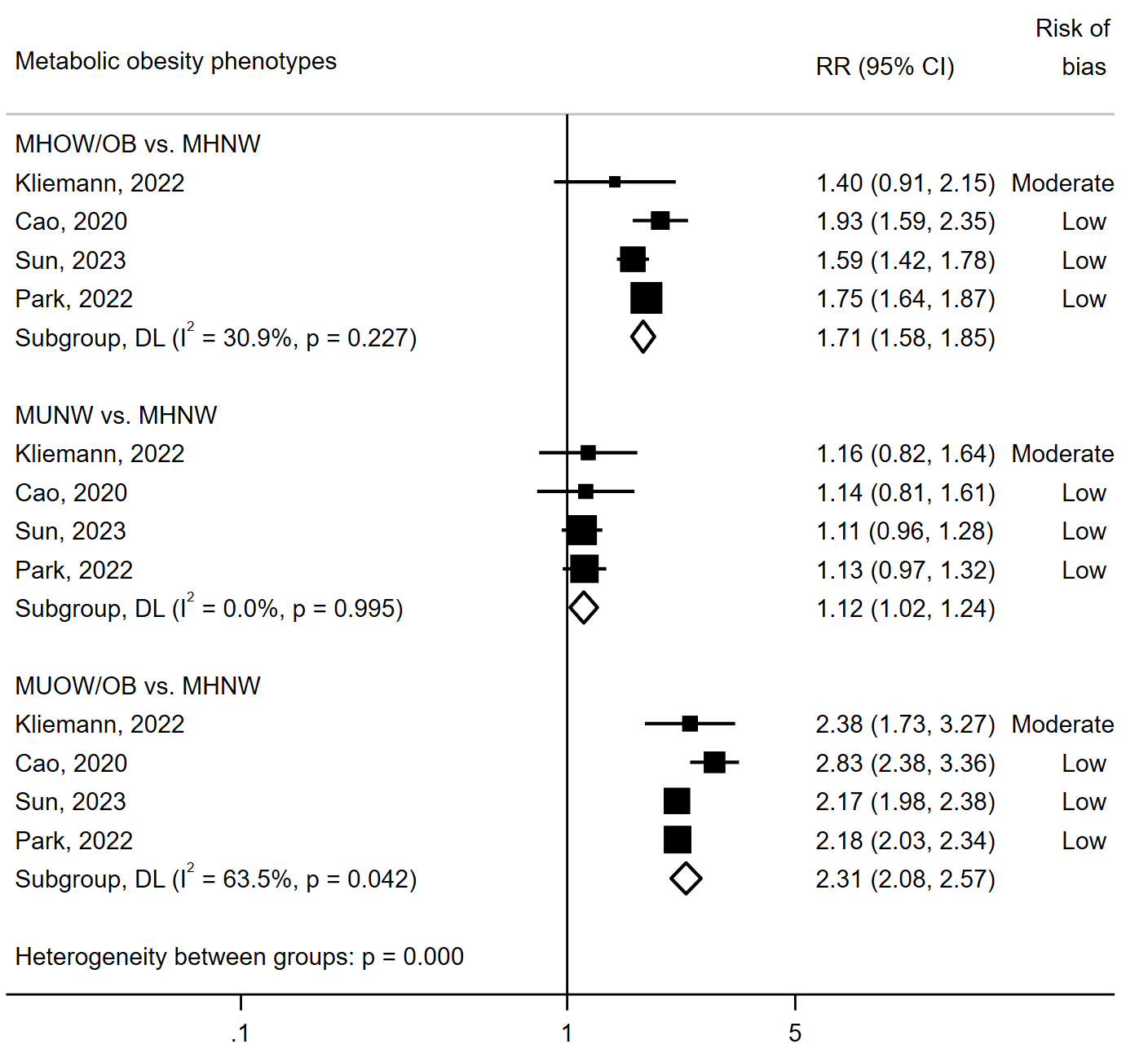


**Figure S7.** Summary estimates for the association between metabolic obesity phenotypes and thyroid cancer risk


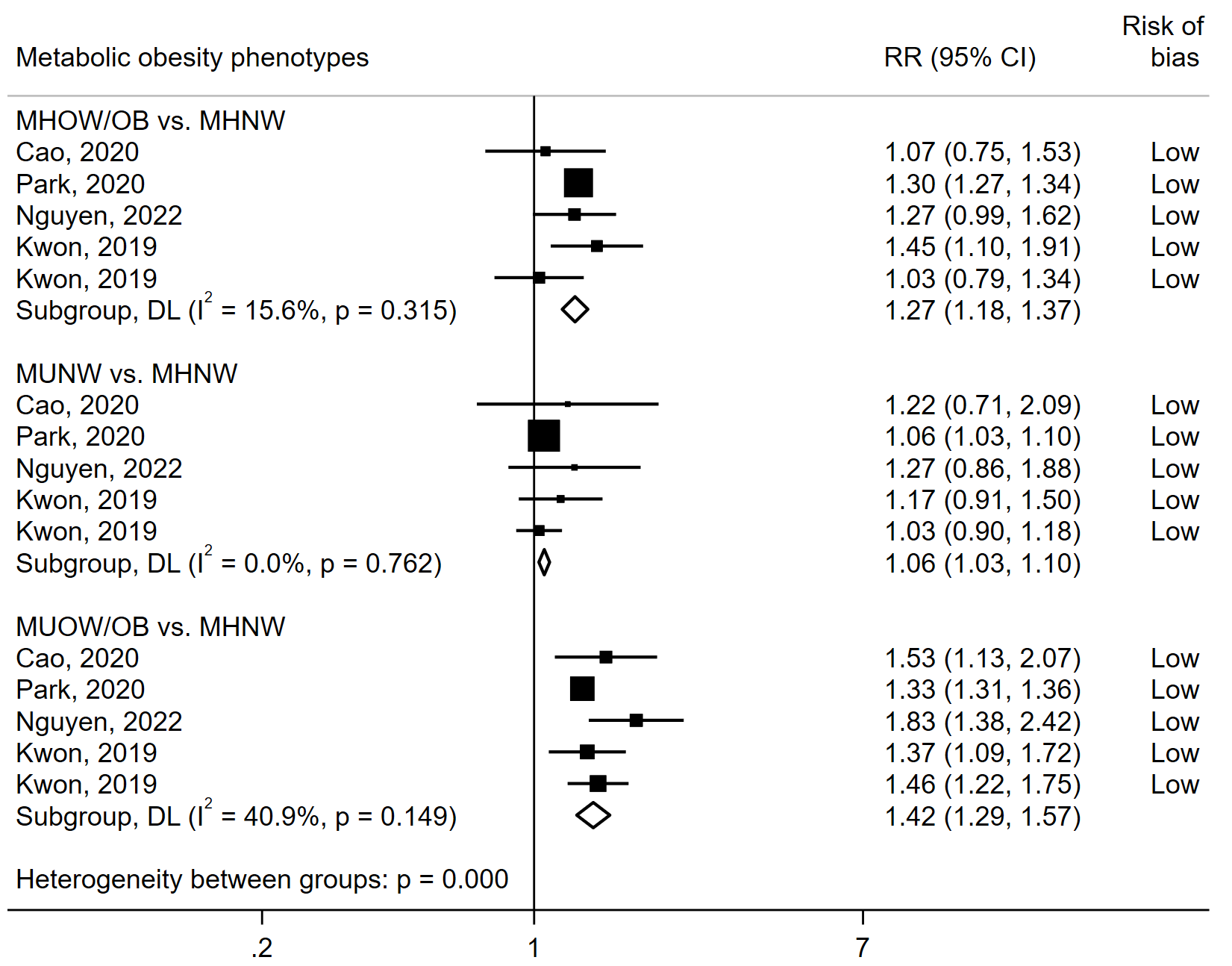


**Figure S8.** Summary estimates for the association between metabolic obesity phenotypes and pancreatic cancer risk


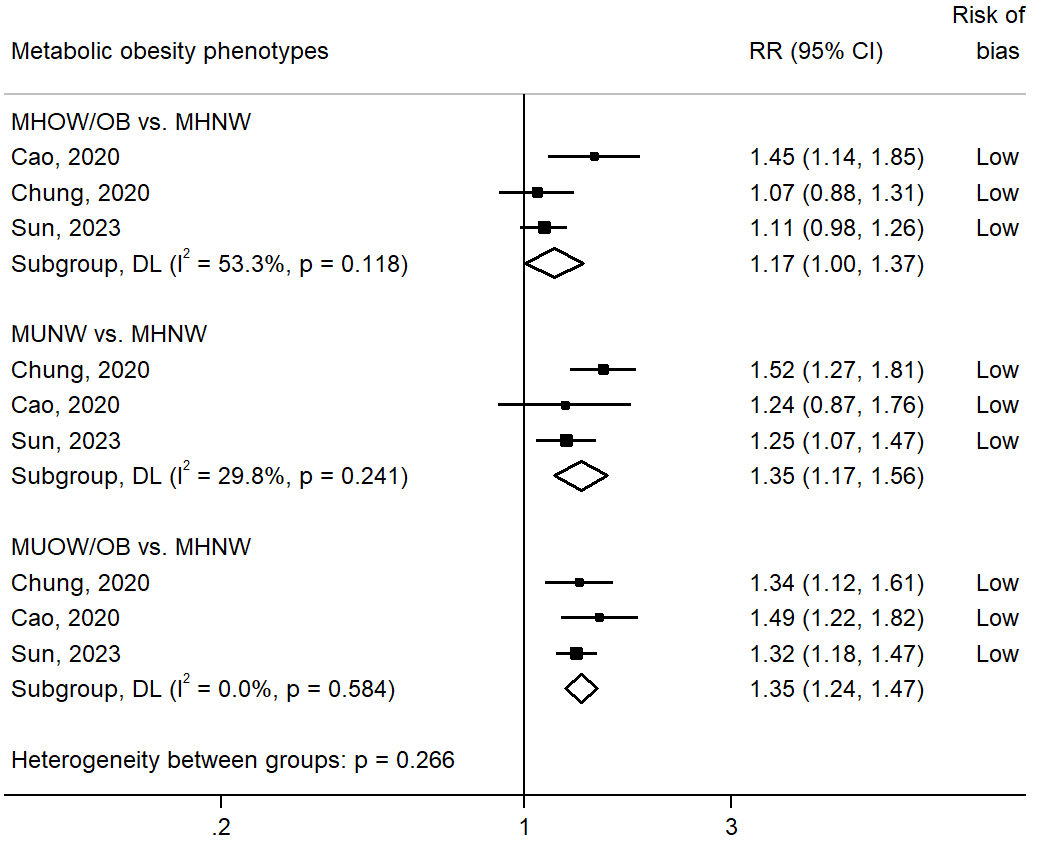


**Figure S9.** Summary estimates for the association between metabolic obesity phenotypes and kidney cancer risk


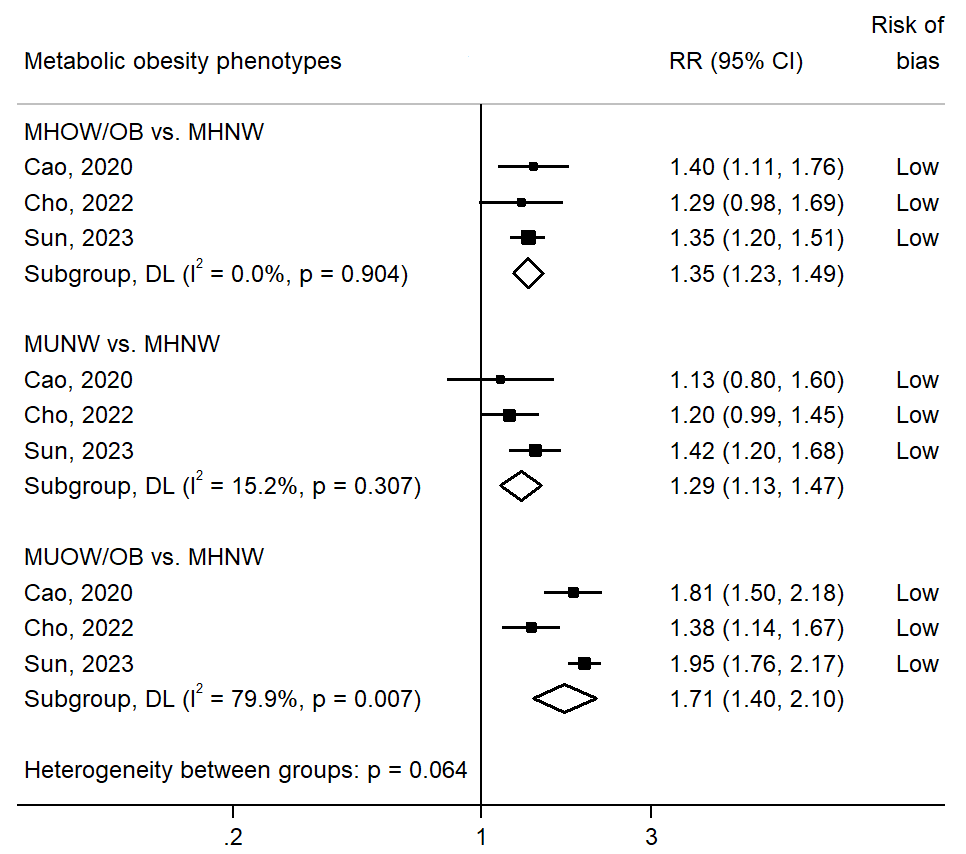


**Figure S10.** Summary estimates for the association between metabolic obesity phenotypes and colon cancer risk


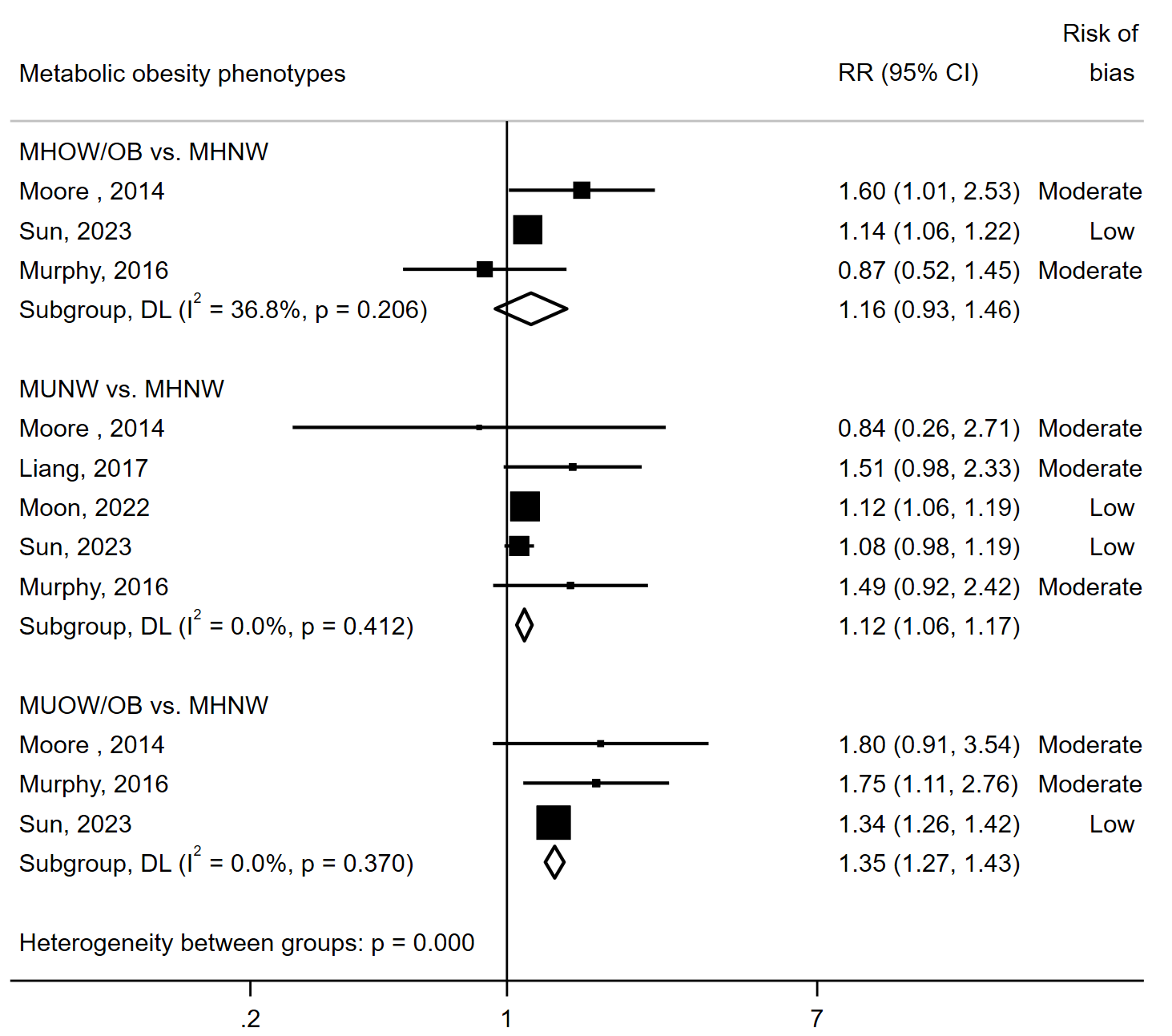


**Figure S11.** Summary estimates for the association between metabolic obesity phenotypes and rectal cancer risk


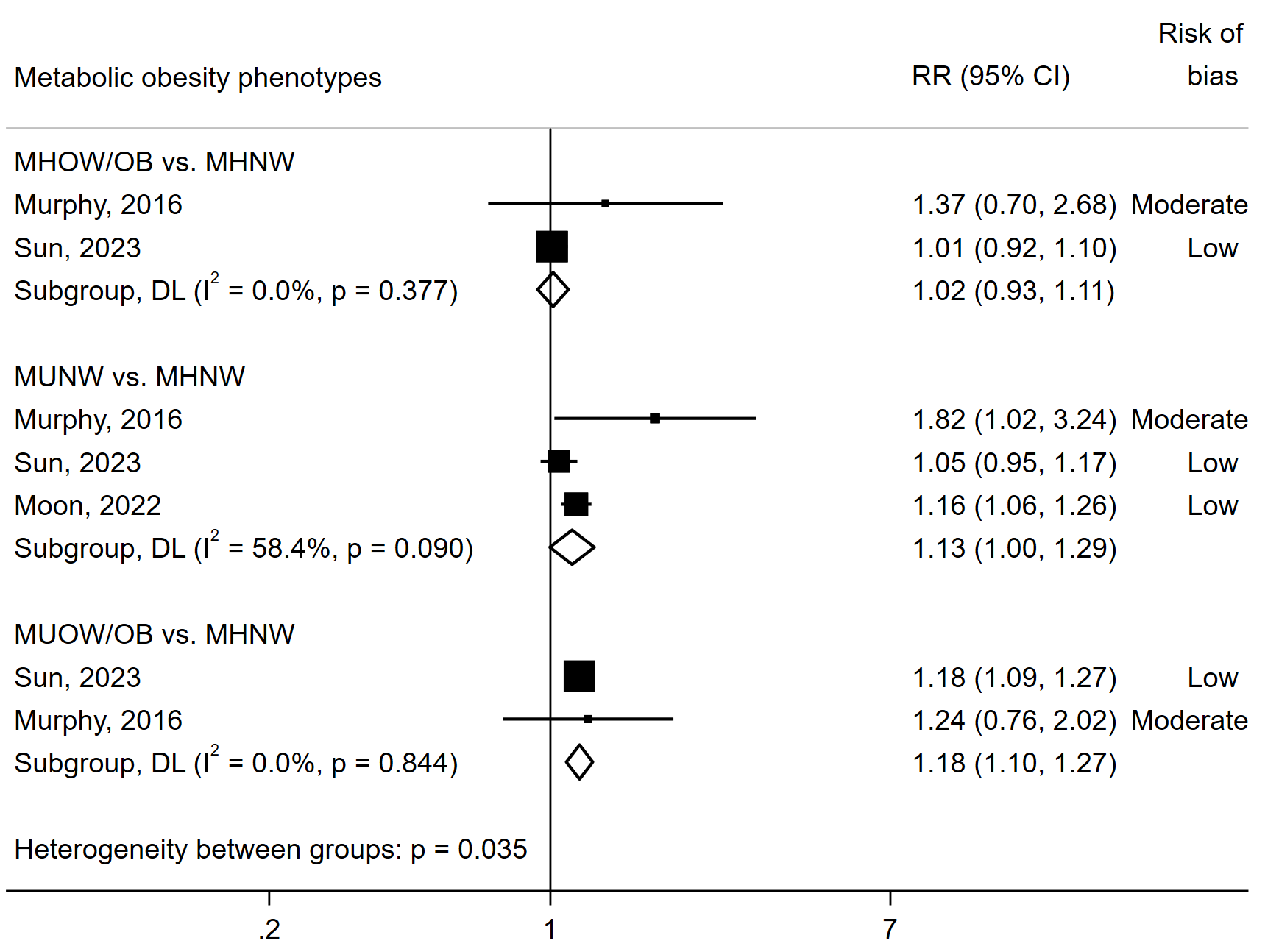


**Figure S12.** Funnel plot for association between MHOW/OB versus MHNW phenotype and postmenopausal breast cancer risk (P_Egger-test_=0.25)

**Figure S13.** Funnel plot for association between MUNW versus MHNW phenotype and postmenopausal breast cancer risk (P_Egger-test_=0.96)

**Figure S14.** Funnel plot for association between MUOW/OB versus MHNW phenotype and postmenopausal breast cancer risk (P_Egger-test_=0.91)

**Figure S15.** Funnel plot for association between MHOW/OB versus MHNW phenotype and colorectal cancer risk (P_Egger-test_=0.88)

**Figure S16.** Funnel plot for association between MUNW versus MHNW phenotype and colorectal cancer risk (P_Egger-test_=0.26)

**Figure S17.** Funnel plot for association between MUOW/OB versus MHNW phenotype and colorectal cancer risk (P_Egger-test_=0.71)

**Figure S18.** Funnel plot for association between MHOW/OB versus MHNW phenotype and endometrial cancer risk (P_Egger-test_=0.67)

**Figure S19.** Funnel plot for association between MUNW versus MHNW phenotype and endometrial cancer risk (P_Egger-test_=0.83)

**Figure S20.** Funnel plot for association between MUOW/OB versus MHNW phenotype and endometrial cancer risk (P_Egger-test_= 0.19)

**Figure S21.** Funnel plot for association between MHOW/OB versus MHNW phenotype and thyroid cancer risk (P_Egger-test_=0.40)

**Figure S22.** Funnel plot for association between MUNW versus MHNW phenotype and thyroid cancer risk (P_Egger-test_=0.34)

**Figure S23.** Funnel plot for association between MUOW/OB versus MHNW phenotype and thyroid cancer risk (P_Egger-test_=0.03)****

**Figure S24.** Influence analysis for the association between MHOW/OB versus MHNW phenotype and postmenopausal breast cancer risk

**Figure S25.** Influence analysis for the association between MUNW versus MHNW phenotype and postmenopausal breast cancer risk

**Figure S26.** Influence analysis for the association between MUOW/OB versus MHNW phenotype and postmenopausal breast cancer risk

**Figure S27.** Influence analysis for the association between MHOW/OB versus MHNW phenotype and colorectal cancer risk

**Figure S28.** Influence analysis for the association between MUNW versus MHNW phenotype and colorectal cancer risk

**Figure S29.** Influence analysis for the association between MUOW/OB versus MHNW phenotype and colorectal risk

**Figure S30.** Influence analysis for the association between MHOW/OB versus MHNW phenotype and endometrial cancer risk

**Figure S31.** Influence analysis for the association between MUNW versus MHNW phenotype and endometrial cancer risk

**Figure S32.** Influence analysis for the association between MUOW/OB versus MHNW phenotype and endometrial cancer risk

**Figure S33.** Influence analysis for the association between MHOW/OB versus MHNW phenotype and thyroid cancer risk

**Figure S34.** Influence analysis for the association between MUNW versus MHNW phenotype and thyroid cancer risk

**Figure S35.** Influence analysis for the association between MUOW/OB versus MHNW phenotype and thyroid cancer risk
