# Supplementary material for: Association of metabolic obesity phenotypes with risk of overall and site-specific cancers: a systematic review and meta-analysis of cohort studies
Source: Br J Cancer. 2024 Sep 24;131(9):1480–95. doi: 10.1038/s41416-024-02857-7 (PMC11519895; doi:10.1038/s41416-024-02857-7)
Supplement: Supplementary file 2 — Supplementary tables [file 41416_2024_2857_MOESM2_ESM.docx]

| **Table S1:** Characteristics of the included 4 publications of associations between metabolic obesity phenotypes and overall cancer risk | | | | | | | | | |
| --- | --- | --- | --- | --- | --- | --- | --- | --- | --- |
| **Author, years,**  **country** | **Study name** | **Study size, age, number of cases** | **Study design** | **Follow-up** | **Metabolically health definition** | **Cancer type** | **Exposure categories** | **Relative risk (95% confidence interval)** | **Adjustment for confounders** |
| Arnlov et al 2010, Sweden | Uppsala Longitudinal Study of Adult Men | 1758 participants without diabetes and aged 50 years, 494 incident cancer cases | Cohort | 30 years | MU was defined as having more than 3 of the following criteria: 1) elevated fasting blood glucose (≥5.6 mmol/l), 2) elevated blood pressure (BP) ≥130/85 mmHg or treatment of hypertension (HT)), 3) Triglycerides (TG) ≥1.7 mmol/l, 4) high-density lipoprotein (HDL) <1.04 mmol/l | Cancer incidence | BMI/MetS-categories MHNW MUNW MHOW MUOW  MHOB MUOB  BMI/insulin resistance  MHNW MUNW MHOW MUOW MHOB MUOB | BMI/MetS-categories  1.00  1.30 (0.82-2.06)  1.09 (0.89-1.33)  1.07 (0.74-1.55) 1.82 (1.04-3.20) 1.73 (1.11-2.70) BMI/insulin resistance 1.00  1.05 (0.80-1.37)  1.15 (0.91-1.44)  1.01 (0.77-1.33)  2.40 (1.31-4.42)  1.56 (1.01-2.41) | Age, smoking status, LDL-cholesterol |
| Lee et al 2022, Republic of Korea | Ansan–Ansung (ASAS) cohort of the Korean Genome and Epidemiology Study (KoGES) | 7530 adults aged ≥ 40 years, 342 cancer cases | Prospective cohort study | 17.4 | MU was defined by the presence of any of the three metabolic health risks (1–3 vs. 0): 1) diabetes mellitus, 2) hypertension, or 3) dyslipidemia | Cancer incidence | MHNW MUNW MHOW/OB MUOW/OB | 1.00 0.94 (0.69–1.29) 0.88 (0.65–1.20) 1.00 (0.75–1.33) | Age, sex, residence area, education level, household income, smoking status, drinking status, and the metabolic equivalent of the task |
| Lin et al 2021, Taiwan | Taiwan National Health Interview Survey | 5734 Taiwanese adults, age ≥20, 428 cancer cases | Cohort | 13.7 years | Participants with healthy cardiometabolic blood profiles included in the metabolic syndrome criteria and an absence of hypertension, diabetes, and hyperlipidemia were considered MH. | Cancer incidence | MHUW  MHNW  MHOW MHO  MUUW  MUNW  MUOW  MUO | 0.63 (0.23, 1.72)  1.00 1.39 (0.90, 2.13)  1.07 (0.51, 2.22)  0.92 (0.42, 2.01)  1.17 (0.87, 1.58)  1.42 (1.05, 1.93)  1.20 (0.84, 1.71) | Sex and age, smoking status, alcohol use, regular exercise, marital status, education, average monthly income |

| **Table S2:** Characteristics of the included 3 publications of associations between metabolic obesity phenotypes and obesity-related cancer risk | | | | | | | | | |
| --- | --- | --- | --- | --- | --- | --- | --- | --- | --- |
| **Author, years,**  **country** | **Study name** | **Study size, age, number of cases** | **Study design** | **Follow-up** | **Metabolically health definition** | **Cancer type** | **Exposure categories** | **Relative risk (95% confidence interval)** | **Adjustment for confounders** |
| Sun et al 2023, Europe | The Metabolic Syndrome and Cancer Project (Me-Can) 2.0 (pooled analysis of 6 cohorts from Norway, Sweden, and Austria) | 797 193 individuals in the study had a mean baseline age of 42.8 years; 23 630 cases of obesity-related cancer | 6 cohorts from Norway, Sweden, and Austria | 19.7 years | MU was defined as the top tertile of the metabolic score, which comprises mid blood pressure [(systolic blood pressure + diastolic blood pressure)/2], plasma glucose, and triglycerides | Obesity-related cancer | Women  MHNW  MUNW MHOW  MUOW MHOB MUOB  Men  MHNW  MUNW MHOW  MUOW MHOB MUOB | Women  1.00  1.09 (1.03–1.14)  1.14 (1.08–1.20)  1.15 (1.10–1.21)  1.34 (1.22–1.47)  1.43 (1.35–1.51)  Men  1.00  1.17 (1.08–1.27)  1.17 (1.10–1.25)  1.38 (1.29–1.48)  1.40 (1.21–1.63)  1.91 (1.74–2.09) | Sex, baseline age, and smoking status, and pack-years and stratified by cohort and date of birth |
| Cao et al 2020, UK | UK Biobank | 390,575 individuals (37–73 years old) from the UK Biobank, 16, 237 incident obesity-related cancer cases | Cohort | 7.8 years | MU was defined as the presence of metabolic syndrome based on the criteria of the Adult Treatment Panel III. MU was considered as having ≥2 of the 4 criteria: 1) elevated BP or the use of antihypertensive medication at baseline, 2) hypertriglyceridemia or current use of lipid-lowering medication at baseline, 3) low HDL cholesterol, 4) hyperglycaemia, defined as fasting blood glucose ≥5.6 mmol/L or use of medications for diabetes at baseline | Obesity-related cancer (calculated by combining risk estimate of adiposity related-cancer types) | MHNW MHOW MHOB MUNW MUOW MUOB  MHOW/OB  MUOW/OB | 1.00  1.07 (1.01–1.13)  1.24 (1.15–1.34)  1.05 (0.98–1.12)  1.14 (1.08–1.20)  1.20(1.14–1.27)  1.12 (1.07–1.17)  1.20 (1.16–1.24) | Sex, age, ethnicity, Townsend deprivation index, qualification, employment status, alcohol intaking, smoking status |
| Moore et al 2014, USA | Framingham Heart Study adults | 3,763 men and women aged 55 to 69 years; 385 obesity-related cancer cases | Prospective cohort study | 7 years | MU phenotype was defined as having elevated blood glucose (> 125 mg/dL) | Obesity-Related Cancer | Metabolic health/ BMI definition MHNW MHOW/OB MUNW MUOW/OB Metabolic health/ BMI definition  MHNW MHOW/OB MUNW MUOW/OB | Metabolic health/ BMI definition 1.00  0.80 (0.40–1.60)  1.60 (1.20–2.10)  2.20 (1.40–3.40) Metabolic health/ WHR definition 1.00 0.97 (0.53–1.80)  1.50 (1.10–2.00)  2.00 (1.30–3.20) | Age, sex (in all-subjects models), height, education, alcohol, cigarettes/day, and physical activity |

| **Table S3:** Characteristics of the included 10 publications of associations between metabolic obesity phenotypes and postmenopausal breast cancer risk | | | | | | | | | |
| --- | --- | --- | --- | --- | --- | --- | --- | --- | --- |
| **Author, years,**  **country** | **Study name** | **Study size, age, number of cases** | **Study design** | **Follow-up** | **Metabolically health definition** | **Cancer type** | **Exposure categories** | **Relative risk (95% confidence interval)** | **Adjustment for confounders** |
| Cao et al. 2020, UK | UK Biobank | 390,575 individuals (37–73 years old) from the UK Biobank, 4633 incident breast cancer cases | Cohort | 7.8 years | MU was defined as the presence of metabolic syndrome based on the criteria of the Adult Treatment Panel III. MU was considered as having ≥2 of the 4 criteria: 1) elevated BP or the use of antihypertensive medication at baseline, 2) hypertriglyceridemia or current use of lipid-lowering medication at baseline, 3) low HDL cholesterol, 4) hyperglycaemia, defined as fasting blood glucose ≥5.6 mmol/L or use of medications for diabetes at baseline | Postmenopausal breast cancer | MHNW MHOW MHOB MUNW MUOW MUOB | 1.00  1.15 (1.02–1.29) 1.36 (1.16–1.59) 1.05 (0.91–1.21) 1.21 (1.08–1.36) 1.29 (1.14–1.46) | Sex, age, ethnicity, Townsend deprivation index, qualification, employment status, alcohol intaking, smoking status |
| Dibaba et al 2018, USA | National Institute of Health-American Association of Retired Persons (NIH-AARP) Diet and Health Study | 94,555 female participants aged 50–71 years, 5380 breast cancer cases | Prospective cohort study | 14 years | MU individuals were defined as those having at least one of the following metabolic abnormalities: 1) high WC >88 cm, 2) dyslipidemia or self-reported history of elevated cholesterol level, 3) high BP or self-reported history of hypertension, and 4) self-reported history of diabetes | Breast cancer | Metabolic health/ BMI  0 MetS & NW  1 MetS & NW  2 MetS & NW  3 MetS & NW  4 MetS & NW  MUNW | 1.00 1.15 (1.02–1.29) 1.27 (1.09–1.48) 1.15 (0.75–1.78) 1.16 (0.16–8.40)  1.19 (1.09–1.30) | Age, race, BMI, education, region, physical activity, smoking, marital status, family history of breast cancer, ovary status, hysterectomy, hormonal therapy use, and ovary status BMI interaction |
| Gunter et al. 2015,  USA | Women's Health Initiative (WHI) | Postmenopausal women aged 50 to 79; 497 breast cancer cases and 2,830 subcohort years at enrolment | Case cohort | 8.2 years | Metabolic health status was defined by HOMA-IR or fasting insulin levels with BMI | Postmenopausal breast cancer | HOMA-IR–based definition of metabolic health MHNW MHOW/OB MUNW MUOW/OB  Insulin-based definition of metabolic health MHNW MHOW/OB MUNW MUOW/OB | HOMA-IR–based definition of metabolic health 1.00 0.96 (0.64–1.42) 1.80 (0.88–3.70) 1.76 (1.19–2.60)  Insulin-based definition of metabolic health 1.00  0.96 (0.64–1.42) 2.06 (1.01–4.22) 2.01 (1.35–2.99) | Age, ethnicity, age at menarche and menopause, parity, first-degree relative with breast cancer, education, alcohol consumption, physical activity, which of the two WHI studies each subject was enrolled in and, among those who participated in the clinical trials, which specific clinical trial arm they were assigned to and whether they were a member of the placebo or treatment group. |
| Kabat et al 2017, USA | Women's Health Initiative (WHI) | 20,944 postmenopausal women aged 50 to 79 years; 1,176 breast cancer cases | Prospective cohort study | 15 years | MU was defined by the presence of metabolic syndrome based on the criteria of the Adult Treatment Panel III (having ≥3 of the 5 following criteria): 1) WC ≥88 cm, 2) triglycerides ≥150 mg/dL, 3) HDL-C <50 mg/dL, 4) glucose ≥100 mg/ dL, and 5) systolic/diastolic blood pressure ≥130/85 mmHg or treatment for hypertension | Postmenopausal breast cancer | MHNW MUNW MHOW MUOW MHO MUO | 1.00 0.86 (0.51–1.38) 1.08 (0.90–1.31) 1.17 (0.93–1.47) 1.31 (1.07–1.61) 1.61 (1.34–1.94) | Age, smoking status, packyears of smoking, alcohol intake, physical activity, and age at first birth. |
| Mahamat-saleh et al 2023, Europe | European Prospective Investigation into Cancer and Nutrition (EPIC) | 610 incident postmenopausal breast cancer cases and 1130 matched controls; women mostly aged 35 years | Nested case-control study | 3 years | Based on the distribution of C-peptide concentration amongst the control population and participants were classified as MH if below the first tertile of C-peptide and MU if above the first tertile. | Postmenopausal breast cancer | Metabolic health/ BMI definition MHNW MHOW/OB MHNW MUOW/OB  Metabolic health/ WC definition MHNW MHOW/OB MHNW MUOW/OB  Metabolic health/ WHR definition MHNW MHOW/OB MHNW MUOW/OB | Metabolic health/ BMI definition  1.00  1.17 (0.80–1.72) 1.02 (0.71–1.46) 1.58 (1.14–2.19)  Metabolic health/ WC definition  1.00  1.26 (0.85–1.85) 1.27 (0.88–1.83) 1.51 (1.09–2.08)  Metabolic health/ WHR definition  1.00  0.92 (0.62–1.36) 1.14 (0.81–1.60) 1.29 (0.94–1.77) | Age at blood collection, time of day at blood collection, fasting status at blood collection, age at menarche, age at first full-term pregnancy and parity, age at menopause,  breastfeeding, ever use of contraceptive pills, ever use of menopausal hormonal therapy, physical activity index, alcohol consumption, smoking status,  educational level, height, and energy intake |
| Park et al 2017, USA | The Sister Study | 50884 women, aged 35 to 74 years enrolled from 2003 through 2009 1,388 invasive breast cancer cases | Prospective cohort study | 6.4 years | Subjects with MU were defined as having at least one cardiometabolic abnormality which included: 1) high waist circumference; 2) elevated blood pressure; 3) previously diagnosed diabetes or antidiabetic drug treatment; and 4) cholesterol-lowering medication use | Breast cancer | BC MHNW MUNW MHOW/OB MUOW/OB Postmenopausal MHNW MUNW MHOW/OB MUOW/OB Premenopausal MHNW MUNW MHOW/OB MUOW/OB | Breast cancer  1.00 1.12 (0.92–1.37) 1.14 (0.95–1.37) 1.28 (1.12–1.48) Postmenopausal  1.00 1.26 (1.01–1.56) 1.24 (0.99–1.55) 1.51 (1.28–1.78) Premenopausal 1.00 0.89 (0.48–1.65) 0.94 (0.67–1.32) 0.71 (0.52–0.97) | Age at baseline, race, education, age at menarche, breastfeeding history, age at first live birth, parity, hormone replacement therapy, oral contraceptive use, menopausal status at baseline, sister age at diagnosis of breast cancer, smoking history, alcohol consumption, and physical activity |
| Reeves et al 2012, USA | The Study of Osteoporotic  Fractures (SOF) | 8956 women aged ≥65 enrolled between 1986 and 1988 from four U.S. locations; 551 breast cancer cases | Prospective cohort study | 14.4 years | MU individuals were defined as those having at least one of the following metabolic abnormalities: 1) elevated WC, 2) hypertension, and 3) diabetes | Postmenopausal breast cancer | Metabolic health/ BMI 0 MetS & NW 1 MetS & NW ≥2 MetS & NW 0 MetS & OW/OB 1 MetS & OW/OB ≥2 MetS & OW/OB | 1.00 1.03 (0.75–1.41) 1.28 (0.64–2.57) 1.14 (0.77–1.64) 1.26 (0.94–1.71) 1.35 (1.01–1.82) | Age, current hormone use, and family history of breast cancer |
| Park et al 2021, Republic of Korea | National Health Insurance Service (NHIS), nationwide representative cohort: 2009 and 2010 | 3,095,336 postmenopausal cancer-free women aged 40–79 years; 23584 breast cancer cases | Prospective cohort study | 9 years | The presence of MetS was defined as the presence of ≥3 of the above five components according to the NECP-ATP III: 1) WC ≥80 cm; 2) elevated fasting blood glucose (FBG) levels, defined as fasting plasma glucose levels ≥100 mg/dL; 3) TG levels ≥150 mg/dL; 4) HDL levels <50 mg/dL for women; and 5) elevated BP (systolic BP ≥ 130 mmHg or diastolic BP ≥ 85 mmHg) | Postmenopausal breast cancer | MHNW MUNW MHOW/OB MUOW/OB | 1.00 1.05 (1.01–1.10) 1.25 (1.21–1.30) 1.37 (1.32–1.42) | Age, age at menarche, age at menopause, hormone replacement therapy use after menopause, delivery, duration of breastfeeding, oral contraceptive use, family history of any cancer, drinking frequency per week during the last 1 year, smoking, and physical activity including vigorous physical activity, moderate physical activity, and walking per week |
| Sun et al 2023, Europe | The Metabolic Syndrome and Cancer Project (Me-Can) 2.0 (pooled analysis of 6 cohorts from Norway, Sweden, and Austria) | 797 193 individuals in the study had a mean baseline age of 42.8; 6792 postmenopausal breast cancer cases. | 6 cohorts from Norway, Sweden and Austria | 19.7 years | MU was defined as the top tertile of the metabolic score, which comprises mid blood pressure [(systolic blood pressure + diastolic blood pressure)/2], plasma glucose, and triglycerides | Postmenopausal breast cancer | MHNW  MUNW MHOW  MUOW MHOB MUOB | 1.00  1.04 (0.96–1.12)  1.08 (1.00–1.16)  1.04 (0.97–1.13)  1.13 (0.97–1.31)  1.08 (0.99–1.18) | Sex, baseline age, smoking status, and pack-years and stratified by cohort and date of birth |

| **Table S4:** Characteristics of the included 10 publications of associations between metabolic obesity phenotypes and colorectal cancer risk | | | | | | | | | |
| --- | --- | --- | --- | --- | --- | --- | --- | --- | --- |
| **Author, years,**  **country** | **Study name** | **Study size, age, number of cases** | **Study design** | **Follow-up** | **Metabolically health definition** | **Cancer type** | **Exposure categories** | **Relative risk (95% confidence interval)** | **Adjustment for confounders** |
| Cao et al 2020, UK | UK Biobank | 390,575 individuals (37–73 years old) from the UK Biobank, 2754 incident colorectal cancer cases | Cohort | 7.8 years | MU was defined as the presence of metabolic syndrome based on the criteria of the Adult Treatment Panel III. MU was considered as having ≥2 of the 4 criteria: 1) elevated BP or the use of antihypertensive medication at baseline, 2) hypertriglyceridemia or current use of lipid-lowering medication at baseline, 3) low HDL cholesterol, 4) hyperglycaemia, defined as fasting blood glucose ≥5.6 mmol/L or use of medications for diabetes at baseline | Colorectal | MHNW MHOW MHOB MUNW MUOW MUOB | 1.00  1.12 (0.98–1.27) 1.10 (0.92–1.33) 1.13 (0.97–1.31) 1.19 (1.06–1.34) 1.29 (1.14–1.47) | Sex, age, ethnicity, Townsend deprivation index, qualification, employment status, alcohol intaking, smoking status |
| Cho et al 2020, Republic of Korea | National Health Insurance Service–National Sample Cohort; 2009–2010 and 2011–2012 | 319,397 Korean adults (mean age of 58.8), 6863 colorectal cancer cases | National Sample Cohort | NA | MU was defined as having ≥2 of the following risk factors: 1) systolic BP ≥130 mmHg and/ or diastolic BP ≥85 mmHg and/or taking antihypertensive medications; 2) TG level ≥150 mg/dl and/or taking lipid-lowering medications; 3) FPG level ≥100 mg/dl and/or taking antidiabetic medications; and 4) HDL-C levels <40 mg/ dl in men and <50 mg/dl in women | Colorectal | MHNW MHOW/OB MUNW MUOW/OB | 1.00 1.14 (1.04–1.26)  1.19 (1.12–1.27)  1.21 (1.13–1.29) | Age, sex, income, smoking, alcohol drinking, and presence of IBD. |
| Kabat et al 2018, USA | Women's Health Initiative (WHI) | 25 446 women with biomarkers sample from WHI and aged between 50 and 79 years, 474 colorectal cancer cases | Prospective cohort study | 5.3 years | MU was defined by the presence of metabolic syndrome based on the criteria of the Adult Treatment Panel III - having ≥3 of the 5 following criteria: 1) waist circumference ≥88 cm, 2) triglycerides ≥150 mg/dl, 3) HDL-C <50 mg/dl, 3) glucose ≥100 mg/dl and 4) systolic/diastolic blood pressure ≥130/85 mmHg or treatment for hypertension | Colorectal | MHNW  MUNW  MHOW  MUOW  MHOB  MUOB | 1.00 1.65 (0.99–2.74) 0.80 (0.59–1.08) 1.11 (0.79–1.57) 0.85 (0.60–1.20) 0.97 (0.72–1.32) | Age, smoking status, alcohol intake, physical activity, aspirin intake, dietary calcium intake, dietary folate intake, caloric intake, oral contraceptives, hormone therapy, family history of colorectal cancer in first-degree relative, education, and ethnicity |
| Liang et al 2017, USA | Women's Health Initiative (WHI) | 5,068 postmenopausal women aged 50 and 79 years and with BMI 18.5–<25 kg/m^2^, 114 colorectal cancer cases | Prospective cohort study | 5.3 years | Women with two or more of the four components were classified as MU: 1) elevated triglycerides, 2) low HDL-C, 3) elevated blood pressure, and 4) elevated fasting glucose | Colorectal  Colon | Colorectal  MHNW  MUNW Colon MHNW  MUNW | Colorectal  1.00 1.49 (1.02–2.18) Colon  1.00 1.51 (0.98–2.33) | Age, ethnicity, smoking, alcohol consumption, physical activity, total energy intake, dietary fiber, percent calories from fat, family history of colorectal cancer, NSAIDs use, and treatment arm in each CT |
| Moon et al 2022, Republic of Korea | National Health Insurance Service health examinations in 2009 and 2010 | 6 142 486 Korean adults aged 40-79 year, 27 384 cases of colon cancer and 11 103 cases of rectal cancer | National Sample Cohort | 8.7 years | MU subjects were defined as those having ≥3 of the 5 following criteria: 1) waist circumference ≥88 cm, 2) triglycerides ≥150 mg/dL, 3) HDL-C <50 mg/dL, 4) glucose ≥100 mg/ dL, and 5) systolic/diastolic blood pressure ≥130/85 mmHg or treatment for hypertension | Colon Rectal | Colon MHNW MUNW  MHOW/OB MUOW/OB  Rectal MHNW MUNW  MHOW/OB MUOW/OB | Colon  1.00 1.12 (1.06-1.19)  1.00  1.16 (1.12-1.21)  Rectal  1.00 1.16 (1.06-1.26)  1.00  1.13 (1.06-1.20) | Age, smoking, drinking, vigorous physical activity, moderate physical activity, walking, and family history of cancer, age at menarche, age at menopause, parity, breastfeeding duration, and oral contraceptive use |
| Shin et al 2017, Republic of Korea | National Health Insurance Service–National Sample Cohort. 2008 and 2009 and followed up until 2013 | 408,931 Korean adults, 5108 CRC cases | National Sample Cohort | 9 years | Participants with none of the three metabolic disease components were considered MH: 1) presence of diabetes mellitus, 2) hypertension, and 3) dyslipidemia | Colorectal | MHNW MUNW MHOW/OB MUOW/OB | Women and men  1.00  1.07 (0.98-1.15)  1.12 (1.01-1.24)  1.17 (1.08-1.27)  Women  1.00 1.05 (0.92–1.18) 1.10 (0.94–1.28) 1.08 (0.95–1.23)  Men  1.00  1.11 (1.00-1.24)  1.21 (1.06-1.39)  1.33 (1.19-1.48) | Age, sex, smoking, drinking, exercise, and income |
| Moore et al 2014, USA | Framingham Heart Study adults | 3,763 men and women aged 55 to 69 years; 147 colon cancer cases | Prospective cohort study | 7 years | MU phenotype was defined as having elevated blood glucose (> 125 mg/dL) | Colon | Metabolic health/ BMI definition MHNW MHOW/OB MUNW MUOW/OB | 1.00  0.84 (0.26–2.70)  1.60 (1.00–2.50)  1.80 (0.93–3.60) | Age, sex (in all-subjects models), height, education, alcohol, cigarettes/day, and physical activity |
| Murphy et al 2014, Europe | European Prospective Investigation into Cancer and Nutrition (EPIC) | Participants mostly aged 35 y or above were recruited between 1992 and 2000; 737 colorectal cancer cases and 737 matched controls | Nested case-control study | 3.7 years | Based on the distribution of C-peptide concentration amongst the control population and participants were classified as MH if below the first tertile of C-peptide and MU if above the first tertile | Colorectal  Colon cancer  Rectal cancer | Metabolic health/ BMI definition MHNW MHOW/OB MUNW MUOW/OB  Metabolic health/ WC definition MHNW MHOW/OB MUNW MUOW/OB  Metabolic health/ BMI definition MHNW MHOW/OB MUNW MUOW/OB  Metabolic health/ WC definition MHNW MHOW/OB MUNW MUOW/OB  Metabolic health/ BMI definition MHNW MHOW/OB MUNW MUOW/OB  Metabolic health/ WC definition MHNW MHOW/OB MUNW MUOW/OB | Metabolic health/ BMI definition  1.00  0.96 (0.65–1.42) 1.59 (1.10–2.28)  1.40 (1.01–1.94)  Metabolic health/ WC definition 1.00 1.12 (0.74–1.69)  1.35 (0.95–1.91)  1.66 (1.20–2.28)  Metabolic health/ BMI definition 1.00  0.87 (0.52–1.45)  1.49 (0.92–2.43)  1.75 (1.11–2.77)  Metabolic health/ WC definition  1.00  0.90 (0.53–1.53)  1.18 (0.74–1.88)  2.12 (1.38–3.27)  Metabolic health/ BMI definition 1.00  1.37 (0.70–2.68) 1.82 (1.02–3.23) 1.24 (0.76–2.02)  Metabolic health/ WC definition  1.00  1.71 (0.85–3.44) 1.76 (1.01–3.05) 1.36 (0.82–2.26) | age (±6 mo at recruitment), sex, study centre, follow-up time since blood collection, time of day at blood collection (±4 h), fasting status, menopausal status, phase of menstrual cycle at blood collection, height, smoking status, physical activity, education level, alcohol consumption, and dietary intakes of total energy, red and processed meats, and fibre |
| Sun et al 2023, Europe | The Metabolic Syndrome and Cancer Project (Me-Can) 2.0, (pooled analysis of 6 cohorts from Norway, Sweden, and Austria) | 797 193 individuals in the study had a mean baseline age of 42.8;  2763 colon cancer cases  1485 rectal cancer cases | 6 cohorts from Norway, Sweden and Austria | 19.7 years | MU was defined as the top tertile of the metabolic score, which comprises mid blood pressure [(systolic blood pressure + diastolic blood pressure)/2], plasma glucose, and triglycerides | Colon cancer  Rectal cancer | Women  MHNW  MUNW MHOW  MUOW MHOB MUOB  Men  MHNW  MUNW MHOW  MUOW MHOB MUOB  Women  MHNW  MUNW MHOW  MUOW MHOB MUOB  Men  MHNW  MUNW MHOW  MUOW MHOB MUOB | Women  1.00  1.01 (0.89–1.15)  1.09 (0.96–1.23)  1.18 (1.05–1.33)  1.16 (0.91–1.48)  1.21 (1.05–1.39)  Men  1.00  1.14 (1.01–1.29)  1.13 (1.03–1.25)  1.31 (1.19–1.45)  1.42 (1.12–1.79)  1.85 (1.61–2.12)  Women  1.00  1.14 (0.97–1.33)  1.00 (0.85–1.19)  1.12 (0.95–1.31)  1.32 (0.98–1.76)  1.22 (0.99–1.48)  Men  1.00  0.98 (0.85–1.14)  1.01 (0.90–1.14)  1.16 (1.03–1.31)  0.94 (0.70–1.27)  1.32 (1.11–1.57) | Sex, baseline age, and smoking status, and pack-years and stratified by cohort and date of birth |

| **Table S5:** Characteristics of the included 5 publications of associations between metabolic obesity phenotypes and endometrial cancer risk | | | | | | | | | |
| --- | --- | --- | --- | --- | --- | --- | --- | --- | --- |
| **Author, years,**  **country** | **Study name** | **Study size, age, number of cases** | **Study design** | **Follow-up** | **Metabolically health definition** | **Cancer type** | **Exposure categories** | **Relative risk (95% confidence interval)** | **Adjustment for confounders** |
| Cao et al 2020, UK | UK Biobank | 390,575 individuals (37–73 years old) from the UK Biobank, 734 incident endometrial cancer cases | Cohort | 7.8 years | MU was defined as the presence of metabolic syndrome based on the criteria of the Adult Treatment Panel III. MU was considered as having ≥2 of the 4 criteria: 1) elevated BP or the use of antihypertensive medication at baseline, 2) hypertriglyceridemia or current use of lipid-lowering medication at baseline, 3) low HDL cholesterol, 4) hyperglycaemia, defined as fasting blood glucose ≥5.6 mmol/L or use of medications for diabetes at baseline | Endometrial cancer | MHNW MHOW MHOB MUNW MUOW MUOB | 1.00  1.43 (1.09–1.85) 2.78 (2.08–3.72) 1.14 (0.81–1.61) 1.82 (1.41–2.36) 4.03 (3.20–5.08) | Sex, age, ethnicity, Townsend deprivation index, qualification, employment status, alcohol intaking, smoking status |
| Kliemann et al 2022, Europe | European Prospective Investigation into Cancer and Nutrition (EPIC) | Participants mostly aged 35 y; 817 endometrial cancer cases and 817 controls | Nested case-control study | NA | Based on the distribution of C-peptide concentration amongst the control population and participants were classified as MU if below the first tertile of C-peptide and metabolically unhealthy if above the first tertile | Endometrial cancer | Metabolic health/ BMI definition MHNW MHOW/OB MHNW MUOW/OB  Metabolic health/ WC definition MHNW MHOW/OB MHNW MUOW/OB  Metabolic health/ WHR definition MHNW MHOW/OB MHNW MUOW/OB | Metabolic health/ BMI definition  1.00 1.40 (0.91–2.15)  1.16 (0.82–1.64)  2.38 (1.73–3.27)  Metabolic health/ WC definition  1.00 1.94 (1.24–3.04) 1.48 (1.05–2.10)  2.69 (1.92–3.77)  Metabolic health/ WHR definition  1.00 1.17 (0.75–1.81)  1.68 (1.21–2.35)  1.83 (1.32–2.54) | Study center, fasting status, age at blood collection, time of day at blood collection, menopausal status, exogenous hormone use, phase of menstrual cycle at blood collection, age at menopause, age at menarche, parity, hormone use, physical activity index, smoking status, educational level, alcohol intake, height, energy intake, and diabetes |
| Park et al 2022, Republic of Korea | National Health Insurance Service (NHIS), nationwide representative cohort: 2009 and 2010 | 6,097,686 cancer-free women aged 40–74 years 6,797 endometrial cancer cases | Prospective cohort study | 9 years | The presence of MetS was defined as the presence of ≥3 of the above five components according to the NECP-ATP III: 1) WC ≥80 cm; 2) elevated fasting blood glucose (FBG) levels, defined as fasting plasma glucose levels ≥100 mg/dL; 3) TG levels ≥150 mg/dL; 4) HDL levels <50 mg/dL for women; and 5) elevated BP (systolic BP ≥ 130 mmHg or diastolic BP ≥ 85 mmHg) | Endometrial cancer | MHNW MHOW/OB MUNW MUOW/OB | 1.00 1.75 (1.64–1.87) 1.13 (0.97–1.32) 2.18 (2.03–2.34) | Age, smoking, drinking, vigorous physical activity, moderate physical activity, walking, age at menarche, age at menopause, number of children, breastfeeding, oral contraceptive use, and family history of cancer |
| Sun et al 2023, Europe | The Metabolic Syndrome and Cancer Project (Me-Can) 2.0, (pooled analysis of 6 cohorts from Norway, Sweden, and Austria) | 797 193 individuals in the study had a mean baseline age of 42.8;  2382 endometrial cancer cases | 6 cohorts from Norway, Sweden and Austria | 19.7 years | MU was defined as the top tertile of the metabolic score, which comprises mid blood pressure [(systolic blood pressure + diastolic blood pressure)/2], plasma glucose, and triglycerides | Endometrial cancer | MHNW  MUNW MHOW  MUOW MHOB MUOB | 1.00 1.11 (0.96–1.28)  1.33 (1.17–1.53)  1.46 (1.27–1.67)  2.36 (1.93–2.88)  3.00 (2.65–3.39) | Sex, baseline age, and smoking status, and pack-years and stratified by cohort and date of birth |

| **Table S6:** Characteristics of the included 5 publications of associations between metabolic obesity phenotypes and thyroid cancer risk | | | | | | | | | |
| --- | --- | --- | --- | --- | --- | --- | --- | --- | --- |
| **Author, years,**  **country** | **Study name** | **Study size, age, number of cases** | **Study design** | **Follow-up** | **Metabolically health definition** | **Cancer type** | **Exposure categories** | **Relative risk (95% confidence interval)** | **Adjustment for confounders** |
| Cao et al 2020, UK | UK Biobank | 390,575 individuals (37–73 years old) from the UK Biobank, 212 incident thyroid cancer cases | Cohort | 7.8 years | MU was defined as the presence of metabolic syndrome based on the criteria of the Adult Treatment Panel III. MU was considered as having ≥2 of the 4 criteria: 1) elevated BP or the use of antihypertensive medication at baseline, 2) hypertriglyceridemia or current use of lipid-lowering medication at baseline, 3) low HDL cholesterol, 4) hyperglycaemia, defined as fasting blood glucose ≥5.6 mmol/L or use of medications for diabetes at baseline | Thyroid cancer | MHNW MHOW MHOB MUNW MUOW MUOB | 1.00  0.82 (0.51–1.31) 1.52 (0.89–2.62) 1.22 (0.71–2.08) 1.79 (1.20–2.67) 1.25 (0.79–1.96) | Sex, age, ethnicity, Townsend deprivation index, qualification, employment status, alcohol intaking, smoking status |
| Kwon et al 2019, South Korea | Kangbuk Samsung Health Study | 255,051 men and women aged 18 years or older, 2927 thyroid cancer cases | Prospective cohort study | 5.3 years | MU subjects were defined as those having at least one of the following metabolic abnormalities: 1) fasting glucose level or current use of glucose-lowering agents, 2) BP ≥ 130/85 mmHg or current use of BP-lowering agents, 3) elevated triglyceride level or current use of lipid-lowering agents, 4) low HDL-C, or 5) insulin resistance (HOMA-IR score) | Thyroid cancer | Men MHNW MHOW/OB  MUNW  MUOW/OB  Women MHNW MHOW/OB  MUNW  MUOW/OB | 1.00 1.45 (1.10–1.90)  1.17 (0.92–1.51)  1.37 (1.09–1.72)  1.00 1.03 (0.79–1.34)  1.03 (0.90–1.18)  1.46 (1.22–1.75) | Age, center, year of screening exam, smoking status, alcohol intake, regular exercise, and educational level total cholesterol, HDL-C, triglycerides, glucose, systolic blood pressure, hs-CRP, HOMA-IR, and TSH. |
| Nguyen et al 2022, Republic of Korea. | The Korean Genome and Epidemiology Study - KoGES HEXA cohort | 173,343 participants (age >40 years) enrolled from 2004 to 2013, 471 thyroid cancer cases | Prospective Cohort Study | 4.9 years | Participants with abnormalities in three of these indices were considered MU: 1) triglycerides, 2) blood pressure, 3) HDL-cholesterol, 4) waist circumference (WC), and 5) fasting glucose levels | Thyroid cancer | MHNW MUNW MHOW/OB MUOW/OB | 1.00 1.27 (0.86–1.88) 1.27 (0.99–1.62) 1.83 (1.38–2.42) | Age (years), sex, smoking, alcohol consumption, physical activity, and education |
| Pasqual et al 2023, USA | The Sister Study | 47,739 women aged 35 to 74 residing in the U.S. (including Puerto Rico); 259 thyroid cancer cases | Prospective cohort study | 12.5 years | Obesity-related metabolic conditions were defined as the presence of any of the following: 1) Self-reported dyslipidemia, 2) diabetes type-2, 3) polycystic ovary syndrome, 4) use of anti-cholesterol or antihypertensive medication, or measured systolic blood pressure ≥140 mmHg or diastolic blood pressure ≥90 mmHg | Thyroid cancer | Metabolic health/ BMI MHNW/OW MUNW/OW MHOB MUOB | 1.00 1.74 (1.25–2.42) 1.91 (1.21–3.03) 2.13 (1.49–3.03) | Education, race, smoking, history of thyroid disorders, and healthcare use |
| Park et al 2020, Republic of Korea | Korean National Health Insurance health checkup database | 9,890,917 adults, 77 133 thyroid cancer cases | A Nationwide Population-Based Cohort Study | 7.2 years | The definition of MetS was based on the modified criteria of the National Cholesterol Education Program Adult Treatment Panel III. Individuals with at least three of the following five components were diagnosed with MetS and considered as MU: 1) abdominal obesity, 2) hypertriglyceridemia, 3) low HDL cholesterol levels, 4) elevated blood pressure, and 5) hyperglycemia | Thyroid cancer | MHNW MUNW MHOW/OB MUOW/OB | 1.00 1.06 (1.02–1.09) 1.30 (1.27–1.34) 1.33 (1.30–1.35) | Age, smoking status, alcohol consumption, physical activity, income, and chronic kidney disease |

| **Table S7:** Characteristics of the included 3 publications of associations between metabolic obesity phenotypes and pancreas cancer risk | | | | | | | | | |
| --- | --- | --- | --- | --- | --- | --- | --- | --- | --- |
| **Author, years,**  **country** | **Study name** | **Study size, age, number of cases** | **Study design** | **Follow-up** | **Metabolically health definition** | **Cancer type** | **Exposure categories** | **Relative risk (95% confidence interval)** | **Adjustment for confounders** |
| Chung et al 2020, Republic of Korea | Korean National Health Insurance Service–Health Screening Cohort (NHIS) | 347,434 Korean adults aged ≥40 years, 886 pancreatic cancer cases | A Nationwide Population-Based Cohort Study | 6.1 years | MU was diagnosed using the National Cholesterol Education Program-Adult Treatment Panel III criteria based on the presence of ≥3 of the following factors: 1) fasting glucose levels or the current use of glucose-lowering, 2) BP ≥130/85mm Hg or the use of antihypertensive agents under the ICD-10 codes I10–15; 3) serum TG levels ≥1.7 mmol/L or the current use of lipid-lowering agents under the ICD-10 code E78; 4) HDL levels <1.0 mmol/L (40 mg/dL) in men or <1.3 mmol/L (50 mg/dL) in women or the current use of lipid-lowering agent; and 5) WC >90 cm for men or >85 cm for women | Pancreas cancer | MHNW MUNW MHOW/OB MUOW/OB | 1.00 1.52 (1.27–1.81) 1.07 (0.88–1.31) 1.34 (1.12–1.61) | Age, sex, smoking status, alcohol intake, physical activity, income level, and levels of hemoglobin, creatinine, alanine aminotransferase, and total cholesterol |
| Cao et al 2020, UK | UK Biobank | 390,575 individuals (37–73 years old) from the UK Biobank, 530 incident pancreatic cancer cases | Cohort | 7.8 years | MU was defined as the presence of metabolic syndrome based on the criteria of the Adult Treatment Panel III. MU was considered as having ≥2 of the 4 criteria: 1) elevated BP or the use of antihypertensive medication at baseline, 2) hypertriglyceridemia or current use of lipid-lowering medication at baseline, 3) low HDL cholesterol, 4) hyperglycaemia, defined as fasting blood glucose ≥5.6 mmol/L or use of medications for diabetes at baseline | Pancreas cancer | MHNW MHOW MHOB MUNW MUOW MUOB | 1.00  1.36 (1.01–1.85) 1.61 (1.08–2.42) 1.24 (0.87–1.76) 1.30 (0.98–1.73) 1.73 (1.29–2.32) | Sex, age, ethnicity, Townsend deprivation index, qualification, employment status, alcohol intaking, smoking status |
| Sun et al 2023, Europe | The Metabolic Syndrome and Cancer Project (Me-Can) 2.0 (pooled analysis of 6 cohorts from Norway, Sweden, and Austria) | 797 193 individuals in the study had a mean baseline age of 42.8; 1063 pancreatic cancer cases | 6 cohorts from Norway, Sweden and Austria | 19.7 years | MU was defined as the top tertile of the metabolic score, which comprises mid blood pressure [(systolic blood pressure + diastolic blood pressure)/2], plasma glucose, and triglycerides | Pancreas cancer | MHNW  MUNW MHOW  MUOW MHOB MUOB | 1.00  1.25 (1.07–1.47)  1.08 (0.94–1.24)  1.26 (1.09–1.44)  1.27 (0.96–1.69)  1.43 (1.21–1.72) | Sex, baseline age, and smoking status, and pack-years and stratified by cohort and date of birth |

| **Table S8:** Characteristics of the included 3 publications of associations between metabolic obesity phenotypes and kidney cancer risk | | | | | | | | | |
| --- | --- | --- | --- | --- | --- | --- | --- | --- | --- |
| **Author, years,**  **country** | **Study name** | **Study size, age, number of cases** | **Study design** | **Follow-up** | **Metabolically health definition** | **Cancer type** | **Exposure categories** | **Relative risk (95% confidence interval)** | **Adjustment for confounders** |
| Cho et al 2022, Republic of Korea | Korean National Health Insurance Service-National Health Screening Cohort (NHIS-HEALS) | 514,866 individuals aged between 40 and 79 years, 810 kidney cancer | Cohort | 5.4 years | According to the Adult Treatment Panel III criteria, MH was defined as having no more than one of the risk factors: 1) BP >130/85 mmHg or the use of antihypertensive drugs, 2) TG level >150 mg/dl or the use of lipid-lowering drugs, 3) HDL-cholesterol level <40 mg/dl (men) or 50 mg/dl (women), or 4) FPG level >100 mg/dl or the use of an anti-diabetic treatment | Kidney cancer | MHNW MHOW/OB MUNW MUOW/OB | 1.00 1.29 (0.98–1.69)  1.20 (0.99–1.45)  1.38 (1.14–1.66) | Age, sex, smoking habits, drinking habits, physical activity, and estimated glomerular filtration rate level. |
| Cao et al 2020, UK | UK Biobank | 390,575 individuals (37–73 years old) from the UK Biobank, 682 incident kidney cancer cases | Cohort | 7.8 years | MU was defined as the presence of metabolic syndrome based on the criteria of the Adult Treatment Panel III. MU was considered as having ≥2 of the 4 criteria: 1) elevated BP or the use of antihypertensive medication at baseline, 2) hypertriglyceridemia or current use of lipid-lowering medication at baseline, 3) low HDL cholesterol, 4) hyperglycaemia, defined as fasting blood glucose ≥5.6 mmol/L or use of medications for diabetes at baseline | Kidney cancer | MHNW MHOW MHOB MUNW MUOW MUOB | 1.00  1.23 (0.92–1.65) 1.71 (1.18–2.47) 1.13 (0.80–1.60) 1.51 (1.16–1.97) 2.16 (1.66–2.81) | Sex, age, ethnicity, Townsend deprivation index, qualification, employment status, alcohol intaking, smoking status |
| Sun et al 2023, Europe | The Metabolic Syndrome and Cancer Project (Me-Can) 2.0, which is a pooling of 6 cohorts from Norway, Sweden, and Austria | 797 193 individuals in the study had a mean baseline age of 42.8; 1354 renal cell cancer cases | 6 cohorts from Norway, Sweden and Austria | 19.7 years | MU was defined as the top tertile of the metabolic score, which comprises mid blood pressure [(systolic blood pressure + diastolic blood pressure)/2], plasma glucose, and triglycerides | Kidney cancer | MHNW  MUNW MHOW  MUOW MHOB MUOB | 1.00  1.42 (1.20–1.68)  1.27 (1.11–1.44)  1.59 (1.39–1.83)  1.72 (1.34–2.21)  2.55 (2.18–2.98) | Sex, baseline age, and smoking status, and pack-years and stratified by cohort and date of birth |

| **Table S9:** Characteristics of the included 2 publications of associations between metabolic obesity phenotypes and gastric cancer risk | | | | | | | | | |
| --- | --- | --- | --- | --- | --- | --- | --- | --- | --- |
| **Author, years,**  **country** | **Study name** | **Study size, age, number of cases** | **Study design** | **Follow-up** | **Metabolically health definition** | **Cancer type** | **Exposure categories** | **Relative risk (95% confidence interval)** | **Adjustment for confounders** |
| Hashimoto et al 2020, Japan | NAGALA database (NAfld in Gifu Area, Longitudinal Analysis) study | 19,685 Japanese individuals who received health-checkup programs from 2003 to 2016, 78 gastric cancers | Cohort | 5.5 years | MU was defined as having one or more of these four metabolic factors: 1) fasting plasma glucose, 2) triglycerides, 3) HDL cholesterol and 4) blood pressure | Gastric cancer | MHNW MHOW/OB MUNW MUOW/OB | 1.00 0.69 (0.04–3.39) 1.16 (0.63–2.12) 2.09 (1.10–3.97) | Age, sex, exercise habit, alcohol consumption and pack-year + 1 |
| Cao et al 2020, UK | UK Biobank | 390,575 individuals (37–73 years old) from the UK Biobank, 299 incident stomach cancer cases | Cohort | 7.8 years | MU was defined as the presence of metabolic syndrome based on the criteria of the Adult Treatment Panel III. MU was considered as having ≥2 of the 4 criteria: 1) elevated BP or the use of antihypertensive medication at baseline, 2) hypertriglyceridemia or current use of lipid-lowering medication at baseline, 3) low HDL cholesterol, 4) hyperglycaemia, defined as fasting blood glucose ≥5.6 mmol/L or use of medications for diabetes at baseline | Gastric cancer | MHNW MHOW MHOB MUNW MUOW MUOB | 1.00  1.33 (0.87–2.01) 0.78 (0.39–1.57) 1.07 (0.65–1.76) 1.31 (0.89–1.93) 1.51 (1.01–2.25) | Sex, age, ethnicity, Townsend deprivation index, qualification, employment status, alcohol intaking, smoking status |

| **Table S10:** Characteristics of the included 2 publications of associations between metabolic obesity phenotypes and prostate cancer risk | | | | | | | | | |
| --- | --- | --- | --- | --- | --- | --- | --- | --- | --- |
| **Author, years,**  **country** | **Study name** | **Study size, age, number of cases** | **Study design** | **Follow-up** | **Metabolically health definition** | **Cancer type** | **Exposure categories** | **Relative risk (95% confidence interval)** | **Adjustment for confounders** |
| Cao et al 2020, UK | UK Biobank | 390,575 individuals (37–73 years old) from the UK Biobank, 5073 incident prostate cancer cases | Cohort | 7.8 years | MU was defined as the presence of metabolic syndrome based on the criteria of the Adult Treatment Panel III. MU was considered as having ≥2 of the 4 criteria: 1) elevated BP or the use of antihypertensive medication at baseline, 2) hypertriglyceridemia or current use of lipid-lowering medication at baseline, 3) low HDL cholesterol, 4) hyperglycaemia, defined as fasting blood glucose ≥5.6 mmol/L or use of medications for diabetes at baseline | Prostate cancer | MHNW MHOW MHOB MUNW MUOW MUOB | 1.00  0.96 (0.87–1.05) 0.90 (0.77–1.04) 0.93 (0.83–1.04) 0.97 (0.89–1.06) 0.79 (0.72–0.87) | Sex, age, ethnicity, Townsend deprivation index, qualification, employment status, alcohol intaking, smoking status |
| Kim et al 2019, South Korea | The National Health Insurance System and National Health Checkups databases (NHC databases) | 11,771,252 men who participated in the NHC, aged at average 46.5 years, 56,552 prostate cancer cases | Cohort | 5.4 years | MU was defined as the presence of ≥3 components of metabolic syndrome according to the modified criteria by the National Cholesterol Education Program-Adult Treatment Panel III | Prostate cancer | MHNW MUNW MHOW/OB MUOW/OB | 1.00 1.14 (1.12–1.17) 1.10 (1.07–1.13) 1.25 (1.22–1.28) | Age, smoking, alcohol drinking, exercise, and income |

| **Table S11**: Characteristics of the included 2 publications of associations between metabolic obesity phenotypes and bladder cancer risk | | | | | | | | | |
| --- | --- | --- | --- | --- | --- | --- | --- | --- | --- |
| **Author, years,**  **country** | **Study name** | **Study size, age, number of cases** | **Study design** | **Follow-up** | **Metabolically health definition** | **Cancer type** | **Exposure categories** | **Relative risk (95% confidence interval)** | **Adjustment for confounders** |
| Kim et al 2020, South Korea | The National Health Insurance System and National Health Checkups databases (NHC databases) | 11781768 men who participated in the NHC, aged at average 46.5 years, 17,777 men with bladder cancer | Cohort | 5.4 years | MU was defined as the presence of ≥3 components of metabolic syndrome according to the modified criteria by the National Cholesterol Education Program-Adult Treatment Panel III | Bladder cancer | MHNW MUNW MHOW/OB MUOW/OB | 1.00 1.18 (1.14–1.23) 1.07 (1.02–1.12) 1.31 (1.26–1.36) | Age, smoking, alcohol drinking, exercise, and income |
| Cao et al 2020, UK | UK Biobank | 390,575 individuals (37–73 years old) from the UK Biobank, 523 incident bladder cancer cases | Cohort | 7.8 years | MU was defined as the presence of metabolic syndrome based on the criteria of the Adult Treatment Panel III. MU was considered as having ≥2 of the 4 criteria: 1) elevated BP or the use of antihypertensive medication at baseline, 2) hypertriglyceridemia or current use of lipid-lowering medication at baseline, 3) low HDL cholesterol, 4) hyperglycaemia, defined as fasting blood glucose ≥5.6 mmol/L or use of medications for diabetes at baseline | Bladder cancer | MHNW MHOW MHOB MUNW MUOW MUOB | 1.00  1.18 (0.83–1.66) 1.27 (0.80–2.04) 1.37 (0.94–1.98) 1.35 (0.99–1.83) 1.75 (1.28–2.39) | Sex, age, ethnicity, Townsend deprivation index, qualification, employment status, alcohol intaking, smoking status |

| **Table S12**: Characteristics of the included 2 publications of associations between metabolic obesity phenotypes and liver cancer risk | | | | | | | | | |
| --- | --- | --- | --- | --- | --- | --- | --- | --- | --- |
| **Author, years,**  **country** | **Study name** | **Study size, age, number of cases** | **Study design** | **Follow-up** | **Metabolically health definition** | **Cancer type** | **Exposure categories** | **Relative risk (95% confidence interval)** | **Adjustment for confounders** |
| Cao et al 2020, UK | UK Biobank | 390,575 individuals (37–73 years old) from the UK Biobank, 271 incident liver cancer cases | Cohort | 7.8 years | MU was defined as the presence of metabolic syndrome based on the criteria of the Adult Treatment Panel III. MU was considered as having ≥2 of the 4 criteria: 1) elevated BP or the use of antihypertensive medication at baseline, 2) hypertriglyceridemia or current use of lipid-lowering medication at baseline, 3) low HDL cholesterol, 4) hyperglycaemia, defined as fasting blood glucose ≥5.6 mmol/L or use of medications for diabetes at baseline | Liver | MHNW MHOW MHOB MUNW MUOW MUOB | 1.00  0.95 (0.58–1.54) 1.32 (0.72–2.45) 1.51 (0.93–2.47) 1.24 (0.82–1.89) 1.87 (1.23–2.82) | Sex, age, ethnicity, Townsend deprivation index, qualification, employment status, alcohol intaking, smoking status |
| Sun et al 2023, Europe | The Metabolic Syndrome and Cancer Project (Me-Can) 2.0, which is a pooling of 6 cohorts from Norway, Sweden, and Austria | 797 193 individuals in the study had a mean baseline age of 42.8; 654 liver cancer cases | 6 cohorts from Norway, Sweden and Austria | 19.7 years | MU was defined as the top tertile of the metabolic score, which comprises mid blood pressure [(systolic blood pressure + diastolic blood pressure)/2], plasma glucose, and triglycerides | Liver, intrahepatic bile ducts | MHNW  MUNW MHOW  MUOW MHOB MUOB | 1.00  1.28 (0.98–1.70)  1.14 (0.89–1.46)  1.60 (1.27–2.02)  1.93 (1.28–2.93)  2.74 (2.13–3.53) | Sex, baseline age, and smoking status, and pack-years and stratified by cohort and date of birth |

| **Table S13:** Characteristics of the included 2 publications of associations between metabolic obesity phenotypes and gallbladder risk | | | | | | | | | |
| --- | --- | --- | --- | --- | --- | --- | --- | --- | --- |
| **Author, years,**  **country** | **Study name** | **Study size, age, number of cases** | **Study design** | **Follow-up** | **Metabolically health definition** | **Cancer type** | **Exposure categories** | **Relative risk (95% confidence interval)** | **Adjustment for confounders** |
| Cao et al 2020, UK | UK Biobank | 390,575 individuals (37–73 years old) from the UK Biobank, 59 incident gallbladder cancer cases | Cohort | 7.8 years | MU was defined as the presence of metabolic syndrome based on the criteria of the Adult Treatment Panel III. MU was considered as having ≥2 of the 4 criteria: 1) elevated BP or the use of antihypertensive medication at baseline, 2) hypertriglyceridemia or current use of lipid-lowering medication at baseline, 3) low HDL cholesterol, 4) hyperglycaemia, defined as fasting blood glucose ≥5.6 mmol/L or use of medications for diabetes at baseline | Gallbladder | MHNW MHOW MHOB MUNW MUOW MUOB | 1.00  1.24 (0.52–2.94) 0.63 (0.14–2.88) 0.79 (0.27–2.34) 1.02 (0.45–2.33) 1.31 (0.58–2.98) | Sex, age, ethnicity, Townsend deprivation index, qualification, employment status, alcohol intaking, smoking status |
| Sun et al 2023, Europe | The Metabolic Syndrome and Cancer Project (Me-Can) 2.0 (pooled analysis of 6 cohorts from Norway, Sweden, and Austria) | 797 193 individuals in the study had a mean baseline age of 42.8; 504 gallbladder cancer cases | 6 cohorts from Norway, Sweden, and Austria | 19.7 years | MU was defined as the top tertile of the metabolic score, which comprises mid blood pressure [(systolic blood pressure + diastolic blood pressure)/2], plasma glucose, and triglycerides | Gallbladder | MHNW  MUNW MHOW  MUOW MHOB MUOB | 1.00  1.27 (0.94–1.71)  1.45 (1.12–1.89)  1.37 (1.05–1.79)  1.84 (1.15–2.94)  1.62 (1.17–2.26) | Sex, baseline age, and smoking status, and pack-years and stratified by cohort and date of birth |

| **Table S14:** Characteristics of the included 2 publications of associations between metabolic obesity phenotypes and ovarian cancer risk | | | | | | | | | |
| --- | --- | --- | --- | --- | --- | --- | --- | --- | --- |
| **Author, years,**  **country** | **Study name** | **Study size, age, number of cases** | **Study design** | **Follow-up** | **Metabolically health definition** | **Cancer type** | **Exposure categories** | **Relative risk (95% confidence interval)** | **Adjustment for confounders** |
| Cao et al 2020, UK | UK Biobank | 390,575 individuals (37–73 years old) from the UK Biobank, 488 incident ovarian cancer cases | Cohort | 7.8 years | MU was defined as the presence of metabolic syndrome based on the criteria of the Adult Treatment Panel III. MU was considered as having ≥2 of the 4 criteria: 1) elevated BP or the use of antihypertensive medication at baseline, 2) hypertriglyceridemia or current use of lipid-lowering medication at baseline, 3) low HDL cholesterol, 4) hyperglycaemia, defined as fasting blood glucose ≥5.6 mmol/L or use of medications for diabetes at baseline | Ovarian cancer | MHNW MHOW MHOB MUNW MUOW MUOB | 1.00  1.16 (0.88–1.55) 1.16 (0.78–1.73) 1.20 (0.84–1.71) 1.06 (0.77–1.44) 1.26 (0.93–1.72) | Sex, age, ethnicity, Townsend deprivation index, qualification, employment status, alcohol intaking, smoking status |
| Sun et al 2023, Europe | The Metabolic Syndrome and Cancer Project (Me-Can) 2.0 (pooled analysis of 6 cohorts from Norway, Sweden, and Austria) | 797 193 individuals in the study had a mean baseline age of 42.8; 1614 ovarian cancer cases | 6 cohorts from Norway, Sweden, and Austria | 19.7 years | MU was defined as the top tertile of the metabolic score, which comprises mid blood pressure [(systolic blood pressure + diastolic blood pressure)/2], plasma glucose, and triglycerides | Ovarian cancer | MHNW  MUNW MHOW  MUOW MHOB MUOB | 1.00  1.02 (0.88–1.20)  1.12 (0.96–1.30)  0.96 (0.82–1.14)  1.04 (0.77–1.41)  1.17 (0.97–1.41) | Sex, baseline age, and smoking status, and pack-years and stratified by cohort and date of birth |

| **Table S15:** Characteristics of the included 2 publications of associations between metabolic obesity phenotypes and myeloma risk | | | | | | | | | |
| --- | --- | --- | --- | --- | --- | --- | --- | --- | --- |
| **Author, years,**  **country** | **Study name** | **Study size, age, number of cases** | **Study design** | **Follow-up** | **Metabolically health definition** | **Cancer type** | **Exposure categories** | **Relative risk (95% confidence interval)** | **Adjustment for confounders** |
| Cao et al 2020, UK | UK Biobank | 390,575 individuals (37–73 years old) from the UK Biobank, 370 incident multiple myeloma cases | Cohort | 7.8 years | MU was defined as the presence of metabolic syndrome based on the criteria of the Adult Treatment Panel III. MU was considered as having ≥2 of the 4 criteria: 1) elevated BP or the use of antihypertensive medication at baseline, 2) hypertriglyceridemia or current use of lipid-lowering medication at baseline, 3) low HDL cholesterol, 4) hyperglycaemia, defined as fasting blood glucose ≥5.6 mmol/L or use of medications for diabetes at baseline | Myeloma | MHNW MHOW MHOB MUNW MUOW MUOB | 1.00  0.76 (0.54–1.07) 1.44 (0.96–2.16) 0.57 (0.36–0.90) 0.86 (0.63–1.17) 1.01 (0.73–1.40) | Sex, age, ethnicity, Townsend deprivation index, qualification, employment status, alcohol intaking, smoking status |
| Sun et al 2023, Europe | The Metabolic Syndrome and Cancer Project (Me-Can) 2.0, (pooled analysis of 6 cohorts from Norway, Sweden, and Austria) | 797 193 individuals in the study had a mean baseline age of 42.8; 458 incident multiple myeloma cases | 6 cohorts from Norway, Sweden and Austria | 19.7 years | MU was defined as the top tertile of the metabolic score, which comprises mid blood pressure [(systolic blood pressure + diastolic blood pressure)/2], plasma glucose, and triglycerides | Myeloma | Women  MHNW  MUNW MHOW  MUOW MHOB MUOB  Men  MHNW  MUNW MHOW  MUOW MHOB MUOB | Women  1.00  1.06 (0.78–1.44)  1.04 (0.78–1.39)  0.92 (0.60–1.24)  1.23 (0.74–2.05)  0.83 (0.57–1.20)  Men  1.00  1.00 (0.72–1.37)  1.53 (1.24–1.88)  1.55 (1.24–1.94)  0.68 (0.35–1.36)  0.98 (0.66–1.46) | Sex, baseline age, and smoking status, and pack-years and stratified by cohort and date of birth |

| **Table S16**: Characteristics of the included 1 publication of associations between metabolic obesity phenotypes and lung cancer risk | | | | | | | | | |
| --- | --- | --- | --- | --- | --- | --- | --- | --- | --- |
| **Author, years,**  **country** | **Study name** | **Study size, age, number of cases** | **Study design** | **Follow-up** | **Metabolically health definition** | **Cancer type** | **Exposure categories** | **Relative risk (95% confidence interval)** | **Adjustment for confounders** |
| Shao et al 2022, UK | UK Biobank | 450,482 UK Biobank participants aged 40–69 years, 3654 lung cancer cases | Prospective Cohort Study | 9.1 years | Participants who met 4 of the 6 criteria above were considered MU: 1) systolic/ diastolic blood pressure; 2) C-reactive protein; 3) triacylglycerols; 4) LDL-C and cholesterol-lowering medications; 5) HDL-C; 6) HbA1c and no diabetes medications | Lung cancer | MHUW MUUW MHNW MUNW MHOW MUOW MHOB MUOB | 1.14 (0.64-2.03) 3.24 (1.33-7.87) 1.00 1.04 (0.85-1.28) 0.82 (0.70-0.98) 1.00 (0.86-1.17) 0.76 (0.61-0.95) 0.92 (0.78-1.08) | Age, sex, education level, ethnicity, smoking status, smoking duration, family history of LC, and personal history of emphysema/bronchitis. |

**Footnote:** BMI body mass index; BP blood pressure; CRC colorectal cancer; CI confidence interval; HbA1c haemoglobin; HDL high-density lipoprotein; HOMA-IR homeostasis model assessment for insulin resistance; HT hypertension; FBG fasting blood glucose; LDL low-density lipoprotein; MetS metabolic syndrome; MHNW metabolically healthy normal weight; MHOW/OB metabolically healthy overweight or obese; MHOB metabolically healthy obese; MHOW metabolically healthy overweight; MHUW metabolically healthy underweight; MUNW metabolically unhealthy normal weight; MUOB metabolically unhealthy obese; MUOW metabolically unhealthy overweight; MUOW/OB metabolically unhealthy overweight or obese; MUUW metabolically unhealthy underweight; NECP-ATP III National Cholesterol Education Program Adult Treatment Panel; TG triglycerides; WC waist circumference;

| **Table S17:** Subgroup analyses of association between metabolic obesity phenotypes and postmenopausal breast cancer | | | | | | | | | | | | | | | |
| --- | --- | --- | --- | --- | --- | --- | --- | --- | --- | --- | --- | --- | --- | --- | --- |
|  | **MUNW vs. MHNW** | | | | | **MUOW/OB vs. MHNW** | | | | | **MHOW/OB vs. MHNW** | | | | |
|  | **n** | **SRR (95% CI)** | **I^2^ (%)** | **P_within_*** | **P_between_†** | **n** | **SRR (95% CI)** | **I^2^ (%)** | **P_within_*** | **P_between_†** | **n** | **SRR (95% CI)** | **I^2^ (%)** | **P_within_*** | **P_between_†** |
| **All studies** | 8 | 1.08 (1.03-1.14) | 29.39 | 0.19 |  | 7 | 1.32 (1.17-1.48) | 90.6 | <0.001 |  | 7 | 1.19 (1.12-1.26) | 56.0 | 0.03 |  |
| **MetS component** |  |  |  |  |  |  |  |  |  |  |  |  |  |  |  |
| 1 or more | 4 | 1.18 (1.09-1.28) | 0.0 | 0.69 | 0.02 | 3 | 1.45 (1.28-1.63) | 0.0 | 0.46 | 0.53 | 3 | 1.20 (1.01-1.43) | 0.0 | 0.92 | 0.97 |
| 2 or more | 2 | 1.22 (0.76-1.96) | 52.0 | 0.15 |  | 2 | 1.40 (1.02-1.93) | 64.5 | 0.09 |  | 2 | 1.17 (0.99-1.39) | 24.0 | 0.25 |  |
| 3 or more | 3 | 1.05 (1.01-1.09) | 0.0 | 0.72 |  | 3 | 1.27 (1.04-1.54) | 96.5 | <0.001 |  | 3 | 1.17 (1.06-1.30) | 85.1 | <0.001 |  |
| **Geographical location** |  |  |  |  |  |  |  |  |  |  |  |  |  |  |  |
| North America | 4 | 1.18 (1.09-1.28) | 0.0 | 0.50 | 0.02 | 3 | 1.42 (1.29-1.57) | 0.0 | 0.54 | 0.25 | 3 | 1.19 (1.07-1.33) | 0.0 | 0.91 | 0.17 |
| Europe | 3 | 1.04 (0.97-1.11) | 0.0 | 0.99 |  | 3 | 1.21 (1.03-1.43) | 86.2 | <0.001 |  | 3 | 1.15 (1.05-1.25) | 47.1 | 0.15 |  |
| Asia | 1 | 1.05 (1.01-1.10) | 0.0 | — |  | 1 | 1.37 (1.32-1.42) | — | — |  | 1 | 1.25 (1.21-1.30) | — | — |  |
| **Duration, years** |  |  |  |  |  |  |  |  |  |  |  |  |  |  |  |
| <5 | 1 | 1.02 (0.71-1.46) | — | — | 0.89 | 1 | 1.58 (1.14-2.19) | — | — | 0.47 | 1 | 1.17 (0.80-1.72) |  |  | 0.002 |
| 5-≤10 | 3 | 1.07 (1.00-1.14) | 23.4 | 0.27 |  | 3 | 1.35 (1.24-1.46) | 64.0 | 0.06 |  | 3 | 1.25 (1.21-1.29) | 0.0 | 0.49 |  |
| >10 | 4 | 1.09 (0.99-1.21) | 50.5 | 0.11 |  | 3 | 1.24 (1.00-1.53) | 87.5 | <0.001 |  | 3 | 1.11 (1.05-1.17) | 0.0 | 0.58 |  |
| **Risk of bias** |  |  |  |  |  |  |  |  |  |  |  |  |  |  |  |
| Low | 5 | 1.05 (1.02-1.09) | 0.0 | 0.50 | 0.02 | 5 | 1.30 (1.14-1.48) | 93.6 | <0.001 | 0.61 | 5 | 1.19 (1.11-1.28) | 70.4 | 0.01 | 0.84 |
| Moderate | 3 | 1.17 (1.08-1.27) | 0.0 | 0.59 |  | 2 | 1.38 (1.15-1.64) | 0.0 | 0.32 |  | 2 | 1.15 (0.88-1.51) | 0.0 | 0.92 |  |
| Serious | — | — | — | — |  | — | — | — | — |  | — | — | — | — |  |
| Critical | — | — | — | — |  | — | — | — | — |  | — | — | — | — |  |

**Footnote:** CI confidence interval; MetS metabolic syndrome; MHNW metabolically healthy normal weight; MHOW/OB metabolically healthy overweight or obese; MUNW metabolically unhealthy normal weight; MUOW/OB metabolically unhealthy overweight or obese; SRR summary relative risk;

I^2^ (%) is a measure of the proportion of the heterogeneity attributed to between study variation rather than due to chance. I^2^ values of 25%, 50% and 75% indicate low, moderate and high between-study heterogeneity, respectively.

*P value for heterogeneity within each subgroup

†P value for heterogeneity between subgroups with meta-regression analysis

| **Table S18:** Subgroup analyses of association between metabolic obesity phenotypes and colorectal cancer | | | | | | | | | | | | | | | |
| --- | --- | --- | --- | --- | --- | --- | --- | --- | --- | --- | --- | --- | --- | --- | --- |
|  | **MUNW vs. MHNW** | | | | | **MUOW/OB vs. MHNW** | | | | | **MHOW/OB vs. MHNW** | | | | |
|  | **n** | **RR (95% CI)** | **I^2^ (%)** | **P_within_*** | **P_between_†** | **n** | **RR (95% CI)** | **I^2^ (%)** | **P_within_*** | **P_between_†** | **n** | **RR (95% CI)** | **I^2^ (%)** | **P_within_*** | **P_between_†** |
| **All studies** | 6 | 1.14 (1.06-1.23) | 45.2 | 0.09 |  | 6 | 1.24 (1.16-1.31) | 53.1 | 0.05 |  | 6 | 1.08 (1.00-1.17) | 58.1 | 0.03 |  |
| **MetS component** |  |  |  |  |  |  |  |  |  |  |  |  |  |  |  |
| 1 or more | 2 | 1.49 (1.01-2.18) | 4.0 | 0.31 | 0.49 | 2 | 1.47 (1.09-1.95) | 0.0 | 0.51 | 0.45 | 2 | 1.22 (0.74-2.01) | 63.8 | 0.10 | 0.50 |
| 2 or more | 2 | 1.18 (1.11-1.25) | 0.0 | 0.53 |  | 2 | 1.22 (1.15-1.28) | 0.0 | 0.77 |  | 2 | 1.13 (1.05-1.21) | 0.0 | 0.72 |  |
| 3 or more | 3 | 1.07 (1.02-1.13) | 0.0 | 0.40 |  | 3 | 1.21 (1.10-1.32) | 75.8 | 0.01 |  | 3 | 1.05 (0.95-1.16) | 71.5 | 0.01 |  |
| **Geographical location** |  |  |  |  |  |  |  |  |  |  |  |  |  |  |  |
| North America | 2 | 1.46 (0.87-2.43) | 7.0 | 0.30 | 0.26 | 2 | 1.24 (0.74-2.07) | 57.5 | 0.13 | 0.73 | 2 | 1.11 (0.58-2.13) | 84.7 | 0.01 | 0.70 |
| Europe | 3 | 1.10 (1.01-1.20) | 38.4 | 0.18 |  | 3 | 1.26 (1.17-1.36) | 59.8 | 0.06 |  | 3 | 1.08 (1.01-1.16) | 39.2 | 0.18 |  |
| Asia | 1 | 1.19 (1.12-1.27) |  |  |  | 1 | 1.21 (1.13-1.29) |  |  |  | 1 | 1.14 (1.04-1.25) |  |  |  |
| **Duration, years** |  |  |  |  |  |  |  |  |  |  |  |  |  |  |  |
| <5 | 1 | 1.59 (1.10-2.29) | — | — | 0.06 | 1 | 1.40 (1.01-1.94) |  |  | 0.59 | 1 | 0.96 (0.65-1.42) |  |  | 0.85 |
| 5-≤10 | 4 | 1.18 (1.12-1.26) | 0.0 | 0.50 |  | 4 | 1.21 (1.14-1.28) | 11.6 | 0.33 |  | 4 | 1.08 (0.94-1.25) | 68.0 | 0.02 |  |
| >10 | 1 | 1.07 (1.00-1.14) | — | — |  | 1 | 1.26 (1.11-143) | 84.8 |  |  | 1 | 1.08 (0.96-1.21) | 77.3 | 0.04 |  |
| **Risk of bias** |  |  |  |  |  |  |  |  |  |  |  |  |  |  |  |
| Low | 3 | 1.12 (1.05-1.19) | 44.2 | 0.15 | 0.03 | 3 | 1.24 (1.17-1.32) | 64.4 | 0.04 | 0.96 | 3 | 1.10 (1.04-1.17) | 41.4 | 0.16 | 0.76 |
| Moderate | 3 | 1.55 (1.16-2.06) | 0.0 | 0.57 |  | 3 | 1.25 (0.94-1.67) | 49.1 | 0.14 |  | 3 | 1.04 (0.72-1.50) | 69.4 | 0.04 |  |
| Serious | — | — | — | — |  |  |  |  |  |  |  |  |  |  |  |
| Critical | — | — | — | — |  |  |  |  |  |  |  |  |  |  |  |

**Footnote:** CI confidence interval; MetS metabolic syndrome; MHNW metabolically healthy normal weight; MHOW/OB metabolically healthy overweight or obese; MUNW metabolically unhealthy normal weight; MUOW/OB metabolically unhealthy overweight or obese; SRR summary relative risk;

I^2^ (%) is a measure of the proportion of the heterogeneity attributed to between study variation rather than due to chance. I^2^ values of 25%, 50% and 75% indicate low, moderate and high between-study heterogeneity, respectively.

*P value for heterogeneity within each subgroup

†P value for heterogeneity between subgroups with meta-regression analysis
